# Protective Effects of Policosanol and Atorvastatin Co-Supplementation Against High-Cholesterol and High-Galactose Diet-Induced Metabolic and Organ Dysfunction in Zebrafish

**DOI:** 10.3390/ijms27177687

**Published:** 2026-08-27

**Authors:** Kyung-Hyun Cho, Cheolmin Jeon, Ashutosh Bahuguna, Ji-Eun Kim, Sang Hyuk Lee, Yunki Lee, Seung Hee Baek

**Affiliations:** Raydel HDL Research Institute, Medical Innovation Complex, Daegu 41061, Republic of Korea

**Keywords:** antioxidants, embryo production, fibrosis, intestine, liver steatosis, ovary, paraoxonase, testis, senescence

## Abstract

The study compares the effects of policosanol (Poli), atorvastatin (Ator), and their combination (Poli+Ator) in mitigating high-cholesterol and high-galactose (HCHG) diet-induced metabolic stress and organ damage in zebrafish. After 22 weeks of feeding, the lowest survival probability was observed in the Ator-supplemented group (0.64). In comparison, the Poli and Poli+Ator-supplemented groups showed higher survival probabilities of 0.76 and 0.71, respectively. Compared with Poli, Ator supplementation had a greater effect in mitigating HCHG-induced dyslipidemia. Nonetheless, Poli+Ator demonstrated a significantly greater effect than Ator and Poli in reducing total blood cholesterol (TC), triglycerides (TG), and low-density lipoprotein cholesterol (LDL-C), and in elevating the HDL-C/TC ratio. No significant effect of Ator supplementation was observed on the HCHG-induced elevations in blood glucose and malondialdehyde (MDA) levels or the diminished sulfhydryl content; however, Poli alone and Poli+Ator significantly improved blood glucose, MDA, and sulfhydryl content. Consistently, the non-significant effect of Ator on the amputated tail fin regeneration was substantially improved by the Poli+Ator supplementation. In addition, Poli and Poli+Ator markedly reduce liver steatosis, neutrophil infiltration, and interleukin (IL)-6 production, while inhibiting the generation of oxidative species and senescent-positive cells in the kidney and preventing intestinal fibrosis. Also, Poli and Poli+Ator substantially minimize HCHG-triggered oxidative stress in reproductive organs, enhance spermatozoa count in the testis and substantially improve the embryo-producing ability of zebrafish. The study outlines the beneficial effect of Poli and Poli+Ator in mitigating HCHG-induced dyslipidemia, organ damage, and reproductive health.

## 1. Introduction

Dyslipidemia refers to abnormal blood lipid levels that increase the risk of cardiovascular disease [1], promote inflammation and oxidative stress [2], and contribute to a wide range of adverse health outcomes. Statins are commonly used in medications to counter dyslipidemia [3]. Besides their lipid-lowering effects, statins exert several pleiotropic benefits, including improved endothelial function, antithrombotic effects, and stabilization of atherosclerotic plaques [4]. Among statins, atorvastatin is the most frequently prescribed drug, which competitively inhibits the activity of 3-hydroxy-3-methylglutaryl-coenzyme A (HMG-CoA) reductase, an important enzyme of cholesterol biosynthesis [5]. Despite the beneficial lipid-lowering effect, prolonged statin consumption is linked with impairment in the liver [6,7], brain [7], kidneys [8], and myotoxicity [9], being the most notable adverse effect. Statins in muscle inhibit coenzyme Q_10_ (CoQ_10_) and ATP biogenesis by interfering with mitochondrial function, inducing reactive oxygen species (ROS) production, and disrupting calcium homeostasis, leading to muscle disorders such as rhabdomyolysis and myalgia [10,11,12]. In addition, a notable adverse effect of statins on male reproductive health affecting sperm motility and decreasing intrinsic antioxidant activity has been described [13]. In addition, several recent studies have revealed that statins have an adverse effect on insulin sensitization and secretion [14,15,16], leading to a higher risk of the onset of type 2 diabetes [17,18,19].

In view of the adverse effects associated with stains, a relatively safe alternative is required. As a safe lipid-lowering agent, herbal formulations have attracted substantial attention. Also, the International Lipid Expert Panel has recommended the use of herbal medicines as a lipid-lowering agent, particularly in statin-intolerant individuals [20]. A variety of herbal constituents, including phytoestrogens, phytosterols, policosanol, and red yeast rice, exhibited substantial lipid-lowering effects [21]. In particular, policosanol has demonstrated great potential as a safe lipid-lowering agent [22]. Policosanol is a blend of long-chain aliphatic acids (LCAA), and its composition varies substantially based on the source material and extraction method [22]. For instance, policosanol from Cuban sugarcane wax is a typical mixture of eight LCAA [23] and is well recognized for its cholesterol-lowering effects [22]. In addition, in human studies, policosanol showed a definitive effect on cholesterol efflux capacity, an increase in high-density lipoprotein cholesterol (HDL-C) levels [24], and functional and morphological improvements in HDL [25]. Also, policosanol inhibits platelet aggregation [22], LDL oxidation, and glycation of lipoproteins [23], and lowers blood glucose levels [26]. In recent human studies, policosanol, in combination with exercise, augments HDL-C levels without depleting CoQ_10_ or causing liver toxicity [25]. Based on the promising outcomes of policosanol, more than 25 countries [27] have approved it as a safe natural ingredient for lowering blood cholesterol.

Individually, both policosanol and atorvastatin have been studied for their lipid-lowering effects; however, a comparative analysis of these two, particularly the effects of their combination, has not been extensively studied [28,29,30]. In our preliminary study, policosanol, atorvastatin, and their combination were evaluated at a higher dose (0.1%) for 12 weeks under hyperlipidemic/hyperglycemic conditions [31]. Extending the previous study herein, a long-term effect (22-week) of relatively lower dosage (0.05%) was tested to assess the effects of policosanol (Poli), atorvastatin (Ator), and their combination (Poli+Ator) in mitigating dyslipidemia, hyperglycemia, and oxidative challenge induced by a high-cholesterol and high-galactose diet in zebrafish. Additionally, the effects of Poli, Ator and their combination were assessed in the liver, kidneys, intestine and reproductive health of zebrafish. The rationale of the study was to determine how the effects of the Poli and Ator combination differ from their individual effects and whether this combination could represent a suitable approach for managing metabolic stress and associated adverse events if consumed for a longer duration.

The zebrafish model was chosen as the experimental animal owing to its acceptability as an efficient preclinical model for investigating human diseases, particularly those related to the liver [32], kidney [33], and reproductive organs [34]. Under the influence of a high-cholesterol diet (HCD), zebrafish liver pathophysiology resembles that of human steatosis, with a gene expression profile comparable to that observed in mammalian models [35]. Moreover, zebrafish share many key receptors, proteins, and enzymes involved in lipid metabolism similar to humans. Notably, unlike mice, zebrafish express cholesteryl ester transfer protein (CETP) [36], an important mediator of cholesterol transfer between lipoproteins that plays a key role in human lipoprotein metabolism, highlighting zebrafish suitability as a preclinical model for lipid metabolism and dyslipidemia [36,37]. Based on the abovementioned features, the outcomes from the zebrafish experiments will be helpful in the design and integration of future human studies.

## 2. Results

### 2.1. Comparison of Survival and Body Weight

**Figure 1:**
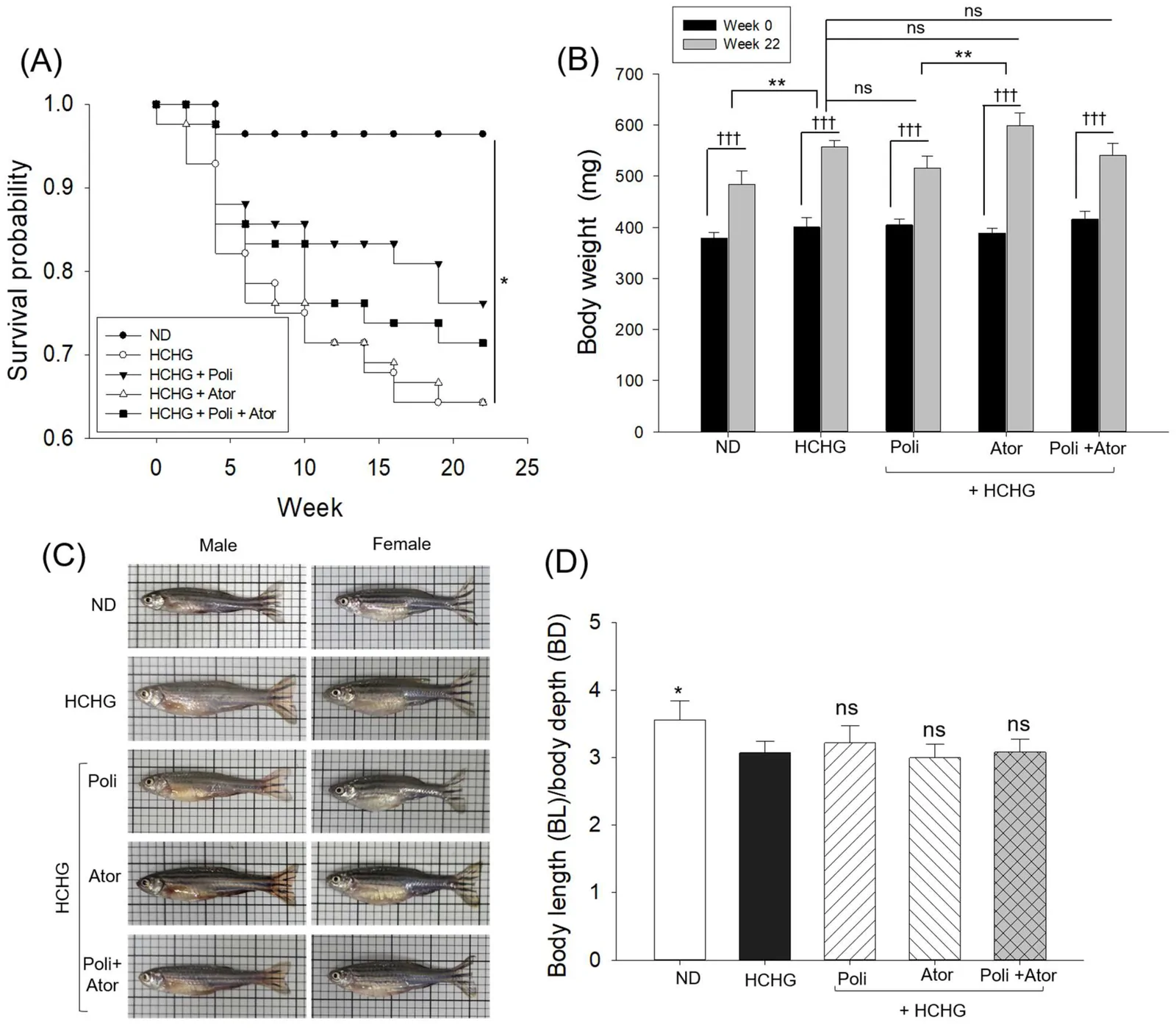
A comparative effect of 22 weeks of feeding different dietary formulations on zebrafish survival and body weight. (**A**) Kaplan–Meier survival curve of zebrafish during 22 weeks of feeding. The symbol * represents a statistical difference at *p* < 0.05 (log-rank *χ*^2^ = 10.3, *p* = 0.036). (**B**) Body weight of zebrafish at the beginning and after 22 weeks of feeding. (**C**) Zebrafish morphology after 22 weeks of feeding. (**D**) Ratio of body length and body depth. Abbreviations: ND, normal diet; HCHG, high-cholesterol and high-galactose diet; Poli, policosanol; and Ator, atorvastatin. * (*p* < 0.05) and ** highlight the statistical significance compared to the HCHG group examined by one-way ANOVA followed by Tukey’s post hoc analysis, while ^†††^ (*p* < 0.001) highlights the statistical difference between the marked groups determined by a paired *t*-test; ns indicates a non-significant difference between the groups.

The Kaplan–Meier survival probability curve, as depicted in Figure 1A, revealed a substantial difference (log-rank *χ*^2^ = 10.3, *p* = 0.036) in survival of zebrafish across the groups. Over 22 weeks of feeding, the highest survival probability (0.96) was observed in the ND (control) group, whereas in the HCHG group, zebrafish survival probability declined in a time-dependent manner. After week 6 of HCHG feeding, the survival probability reached 0.82, then declined to 0.71 by week 12 and ultimately to 0.64 by week 22 (Figure 1A). In contrast, the higher survival probability (0.76) was observed in the Poli+HCHG group at 22 weeks of feeding. The zebrafish in the Ator+HCHG group followed a nearly similar survival probability pattern to that observed in the HCHG group and ended with a survival probability of 0.64 after 22 weeks of feeding. However, zebrafish fed with Poli+Ator showed a markedly higher survival probability of 0.71 after 22 weeks of feeding.

The body weight (BW) among all groups was significantly changed after 22 weeks of feeding as compared to the BW of week 0 (Figure 1B). However, the most noticeable changes, with 54% BW enhancement compared to the week 0 BW, were observed in the Ator-supplemented group, followed by 39% BW enhancement in the HCHG group. In the Poli-supplemented group, a 27% BW enhancement was observed relative to the BW of week 0, and the changes were somewhat similar to the BW changes observed in the ND (control) group. Compared to the ND group, a significantly 16% higher BW was observed in the HCHG group after 22 weeks of feeding (Figure 1B). In contrast, the BW (after 22 weeks of feeding) in the Poli-, Ator-, and Poli+Ator-supplemented groups was statistically unchanged compared to the BW of the HCHG group.

A similar trend was observed in the morphological analysis, quantified by the body length (BL)/body depth (BD) ratio, which revealed a significantly higher BL/BD ratio in the ND group than in the HCHG group (Figure 1C,D). Also, no significant change in the BL/BD ratio was observed in the Poli-, Ator-, and Poli+Ator-supplemented groups compared to the HCHG group.

### 2.2. Organ Morphology and Weight

**Figure 2:**
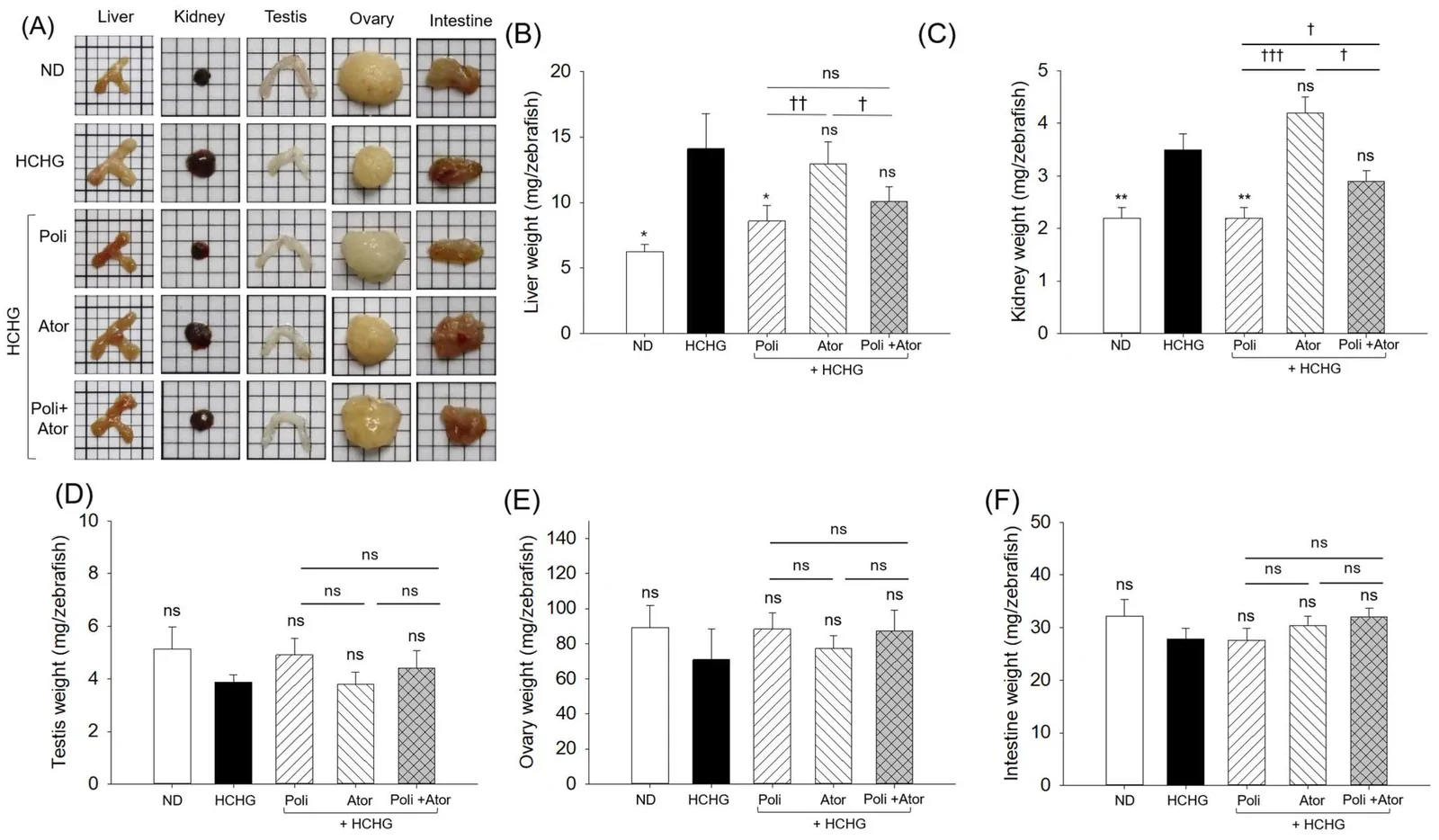
Organ morphology (**A**) and organ weight (**B**–**F**) of zebrafish after 22 weeks of feeding different dietary formulations. Abbreviations: ND, normal diet; HCHG, high-cholesterol and high-galactose diet; Poli, policosanol; and Ator, atorvastatin. * and ** underscore the statistical difference at *p* < 0.05 and *p* < 0.01 between the groups relative to the HCHG group, while ^†^ (*p* < 0.05), ^††^ (*p* < 0.01) and ^†††^ (*p* < 0.001) depict the statistical difference between the marked groups determined by one-way ANOVA, followed by Tukey’s post hoc analysis; ns indicates a non-significant difference between the groups.

As depicted in Figure 2A–C, enhanced liver and kidney size and weight were observed in the HCHG-consuming group, which accounted for 2.2-fold and 1.6-fold higher weight, respectively, than the normal weight of the respective organs detected in the ND (control) group. The HCHG-distorted liver and kidney morphology and elevated weight were substantially reduced by the co-supplementation of Poli, by 1.6-fold and 1.2-fold, respectively. In contrast, no significant effect of Ator was observed on HCHG-induced changes in liver and kidney morphology and weight. However, Poli+Ator substantially reduced HCHG-induced liver and kidney weights.

Unlike liver and kidney weight, a non-significant effect of HCHG was noticed on the alteration of testis, ovary and intestine weights compared to the ND (control) group (Figure 2A,D–F). However, morphological analysis revealed changes in the morphology of the testis, ovaries, and intestine in the HCHG group compared with the ND (control) group, which were substantially reversed by consumption of Poli and Poli+Ator.

### 2.3. Blood Lipoprotein Profile

**Figure 3:**
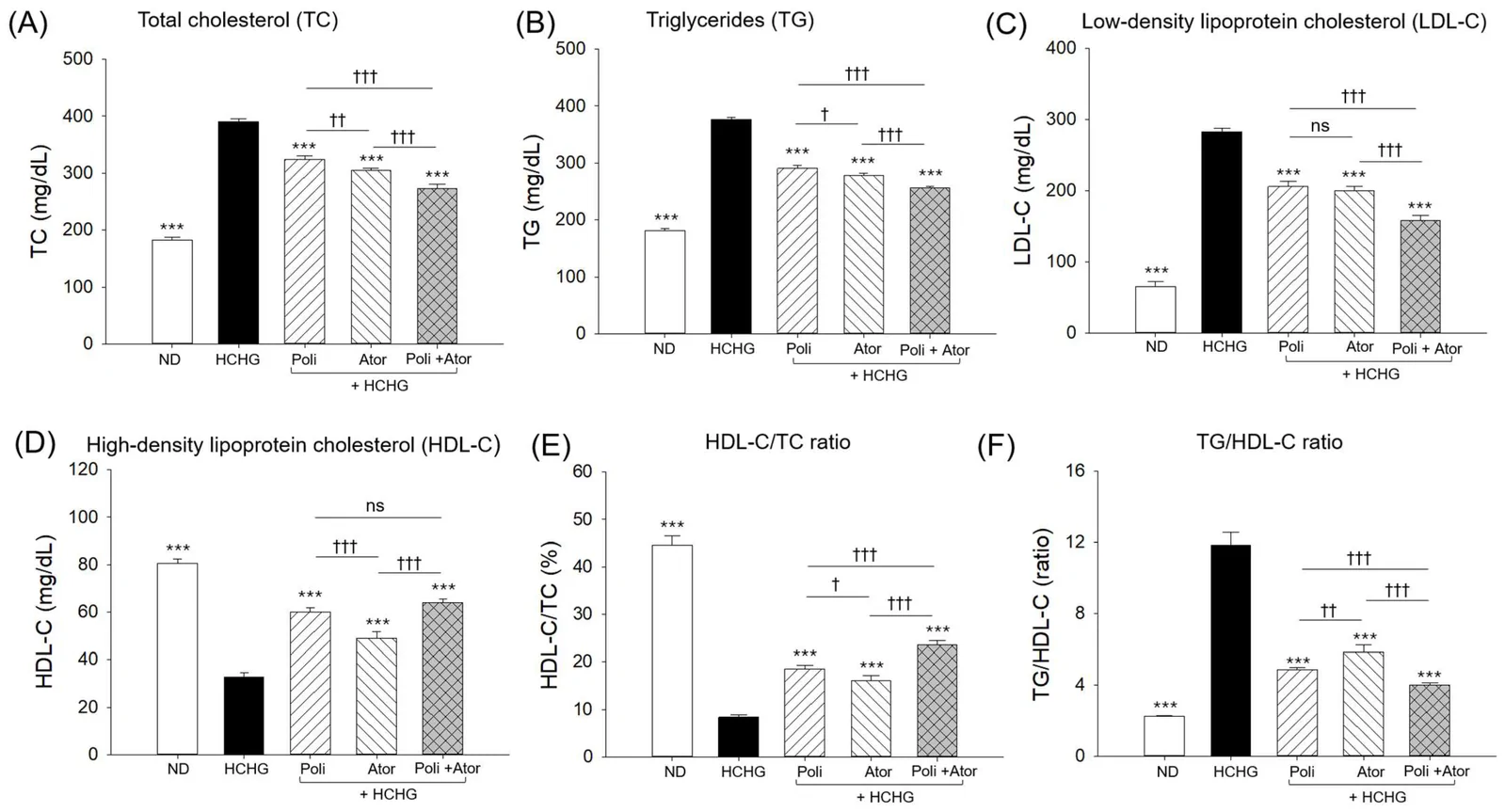
Lipid profile (**A**–**F**) of zebrafish after 22 weeks of feeding the different dietary formulations. Abbreviations: ND, normal diet; HCHG, high-cholesterol and high-galactose diet; Poli, policosanol; and Ator, atorvastatin. Each point in the bar graph represents the mean ± SEM value obtained from three (*n* = 3) independent experiments. *** underscores the statistical difference at *p* < 0.001 between the groups relative to the HCHG group, whereas ^†^ (*p* < 0.05), ^††^ (*p* < 0.01) and ^†††^ (*p* < 0.001) depict the statistical difference between the marked groups evaluated by one-way ANOVA, followed by Tukey’s post hoc analysis; ns represents non-significant difference.

The blood lipoprotein profile of the ND (control) group revealed normal levels of TC, TG, and LDL-C, which were significantly altered following 22-week consumption of HCHG (Figure 3A–C). The supplementation with Poli reduced the HCHG-elevated TC, TG, and LDL-C levels, which were significantly 1.2-fold, 1.3-fold, and 1.4-fold lower than their respective levels detected in the HCHG group. Compared with Poli, Ator supplementation proved more effective in reducing the HCHG-elevated TC, TG and LDL-C levels. However, in the Poli+Ator-supplemented group, significantly lower TC, TG, and LDL-C levels were observed compared to their respective levels in the Poli- and Ator-alone-supplemented groups.

In contrast to the TC, TG, and LDL-C levels, HDL-C was significantly lower in the HCHG group (32.7 mg/dL) than in the ND (control) group (Figure 3D). Supplementation with both Poli and Ator effectively increased HCHG-diminished HDL-C levels to 60.1 mg/dL and 48.9 mg/dL, respectively. Unlike the individual supplementation of Poli and Ator, the Poli+Ator supplementation showed significantly higher HDL-C level. Likewise, Poli+Ator significantly elevated HDL-C/TC (%) compared with the HDL-C/TC (%) observed in the Poli- and Ator-alone-supplemented groups (Figure 3E).

Also, the HCHG-elevated TG/HDL-C ratio was substantially reduced by the individual supplementation of Poli and Ator (Figure 3F). However, in the Poli+Ator-supplemented group, the TG/HDL-C ratio was significantly reduced by 16% and 31% compared to the TG/HDL-C ratio observed in the Poli and Ator individually supplemented groups, respectively. The findings suggest a substantially greater effect of Poli+Ator than of Poli or Ator in mitigating HCHG-induced changes in the plasma lipid profile.

### 2.4. Antioxidant Abilities in Blood

**Figure 4:**
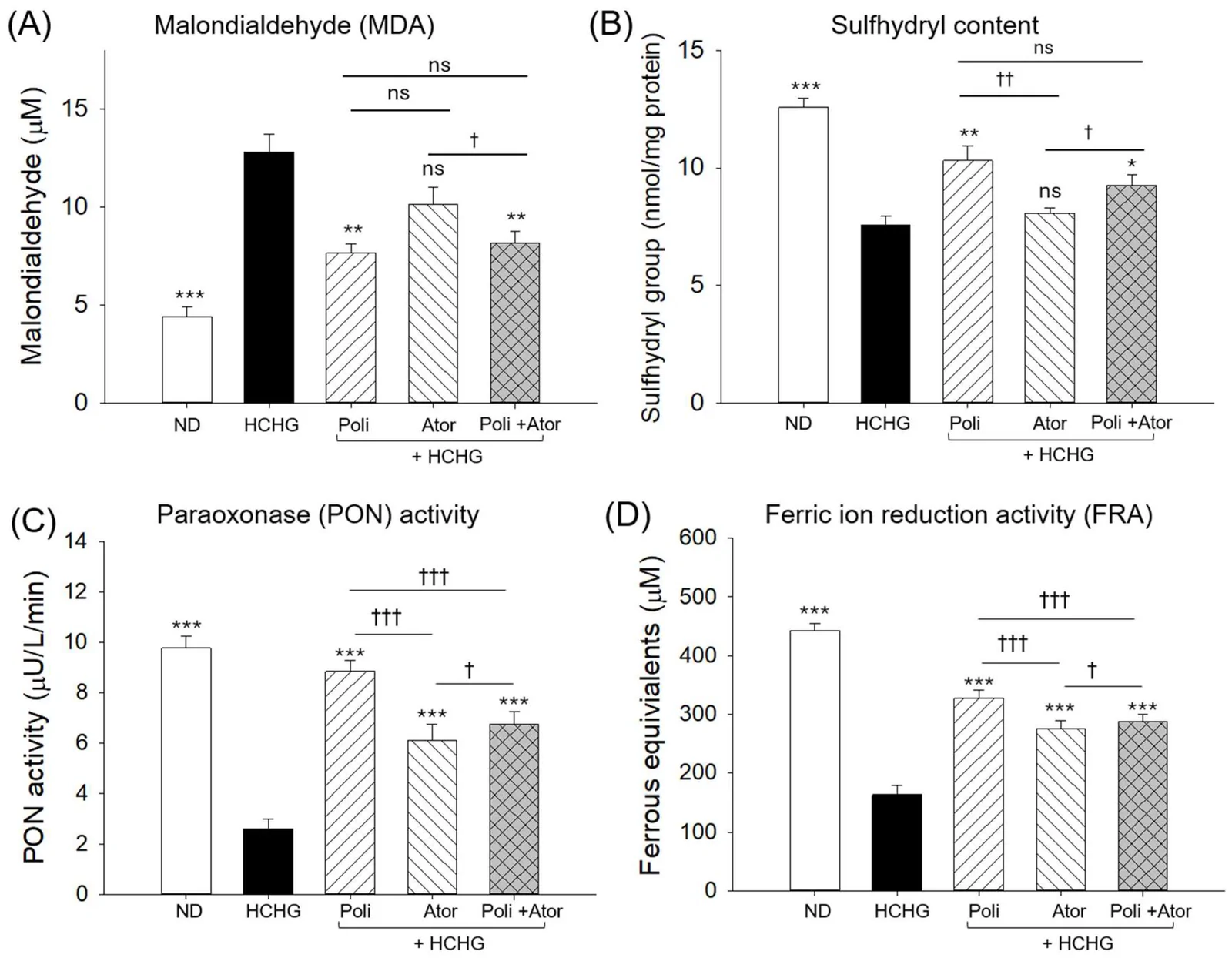
Zebrafish blood (**A**) malondialdehyde (MDA) level, (**B**) sulfhydryl content, (**C**) paraoxonase (PON) activity, and (**D**) ferric ion reduction ability (FRA) after 22 weeks of feeding different dietary formulations. Abbreviations: ND, normal diet; HCHG, high-cholesterol and high-galactose diet; Poli, policosanol; Ator: atorvastatin. Each point in the bar graph represents the mean ± SEM value obtained from three (*n* = 3) independent experiments. *, ** and *** underscore the statistical difference at *p* < 0.05, *p* < 0.01 and *p* < 0.001 between the groups relative to the HCHG group, while ^†^ (*p* < 0.05), ^††^ (*p* < 0.01) and ^†††^ (*p* < 0.001) depict the statistical difference between the marked groups evaluated by one-way ANOVA, followed by Tukey’s post hoc analysis; ns represents non-significant difference.

The MDA level was elevated by 2.9-fold after 22 weeks of HCHG consumption compared with the ND (control) group (Figure 4A). The HCHG-elevated MDA level was significantly lower by 1.7-fold after supplementation with Pol. No significant effect of Ator supplementation was observed on the HCHG-elevated MDA level. However, supplementation of Poli+Ator effectively mitigated the HCHG-induced increase in MDA levels.

The blood sulfhydryl content, PON and FRA activity were significantly reduced by 1.7-fold, 3.8-fold and 2.7-fold by the 22-week intake of HCHG, as compared to their normal values detected in the ND (control) group (Figure 4B–D). Supplementation of Poli substantially elevated the HCHG-diminished sulfhydryl content by 1.4-fold compared to the HCHG group. No significant effect of Ator intake was observed on the enhancement of the HCHG-reduced sulfhydryl content. However, Poli+Ator significantly increased the blood sulfhydryl content by 1.2-fold. Poli supplementation significantly increased PON and FRA activities by approximately 3.4-fold and 2-fold, respectively, compared with the HCHG group. Likewise, PON and FRA activities were 2.3- and 1.7-fold higher in the Ator-supplemented group than in the HCHG group but were significantly lower relative to the Poli-supplemented group. The Poli+Ator-supplemented group displayed a significant ~10% and ~5% elevation of PON and FRA activities compared to the respective values detected in the Ator-alone-supplemented group. The findings highlight a significant beneficial effect of Poli and Poli+Ator in improving HCHG-disturbed plasma oxidative variables and antioxidant parameters.

### 2.5. Tail Fin Regeneration and Blood Glucose Level

**Figure 5:**
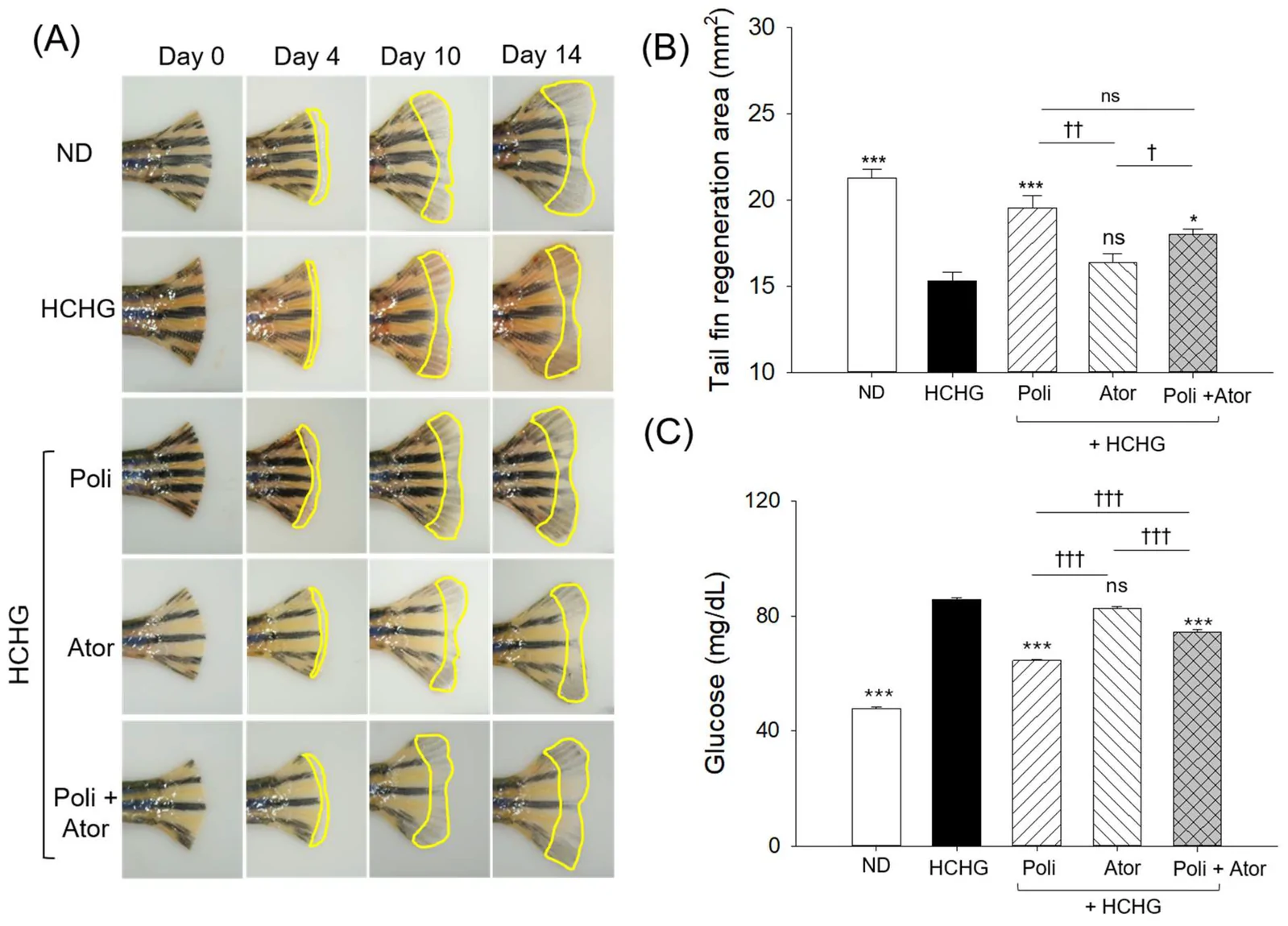
Effect of different dietary formulations on (**A**) tail fin regeneration ability, representative images of zebrafish tail from different groups depicting the regenerated tail after 0, 4, 10 and 14 days post-amputation. Area under the yellow line showed the regenerated tail fin. (**B**) Quantification of the regenerated tail fin area. (**C**) Blood glucose level. Abbreviations: ND, normal diet; HCHG, high-cholesterol and high-galactose diet; Poli, policosanol; Ator: atorvastatin. Each point in the bar graph represents the mean ± SEM value obtained from three (*n* = 3) independent experiments. * and *** underscore the statistical difference at *p* < 0.05 and *p* < 0.001 between the groups relative to the HCHG group, while ^†^ (*p* < 0.05), ^††^ (*p* < 0.01) and ^†††^ (*p* < 0.001) depict the statistical difference between the marked groups evaluated by one-way ANOVA, followed by Tukey’s post hoc analysis; ns represents non-significant difference.

At 14 days post-tail fin amputation, the HCHG group showed the lowest tail fin regeneration ability, which was substantially improved by the supplementation of Poli and Poli+Ator (Figure 5A,B). Contrary to this, Ator supplementation did not significantly improve HCHG-impaired tail fin regeneration.

Compared to the normal blood glucose level in the ND (control) group, a significant 1.8-fold elevation in the blood glucose level was observed in the HCHG-supplemented group, which was substantially reduced by the supplementation of the Poli (Figure 5C). In contrast, no significant effect of Ator was observed on lowering blood glucose levels. However, in the Poli+Ator-supplemented group, a significant ~1.2-fold reduction in blood glucose levels was observed compared with the HCHG- and Ator-supplemented groups. The findings suggest a beneficial effect of Poli and Poli+Ator in enhancing HCHG-compromised tail fin regeneration and reducing elevated blood glucose levels.

### 2.6. Histological Evaluation of Liver

**Figure 6:**
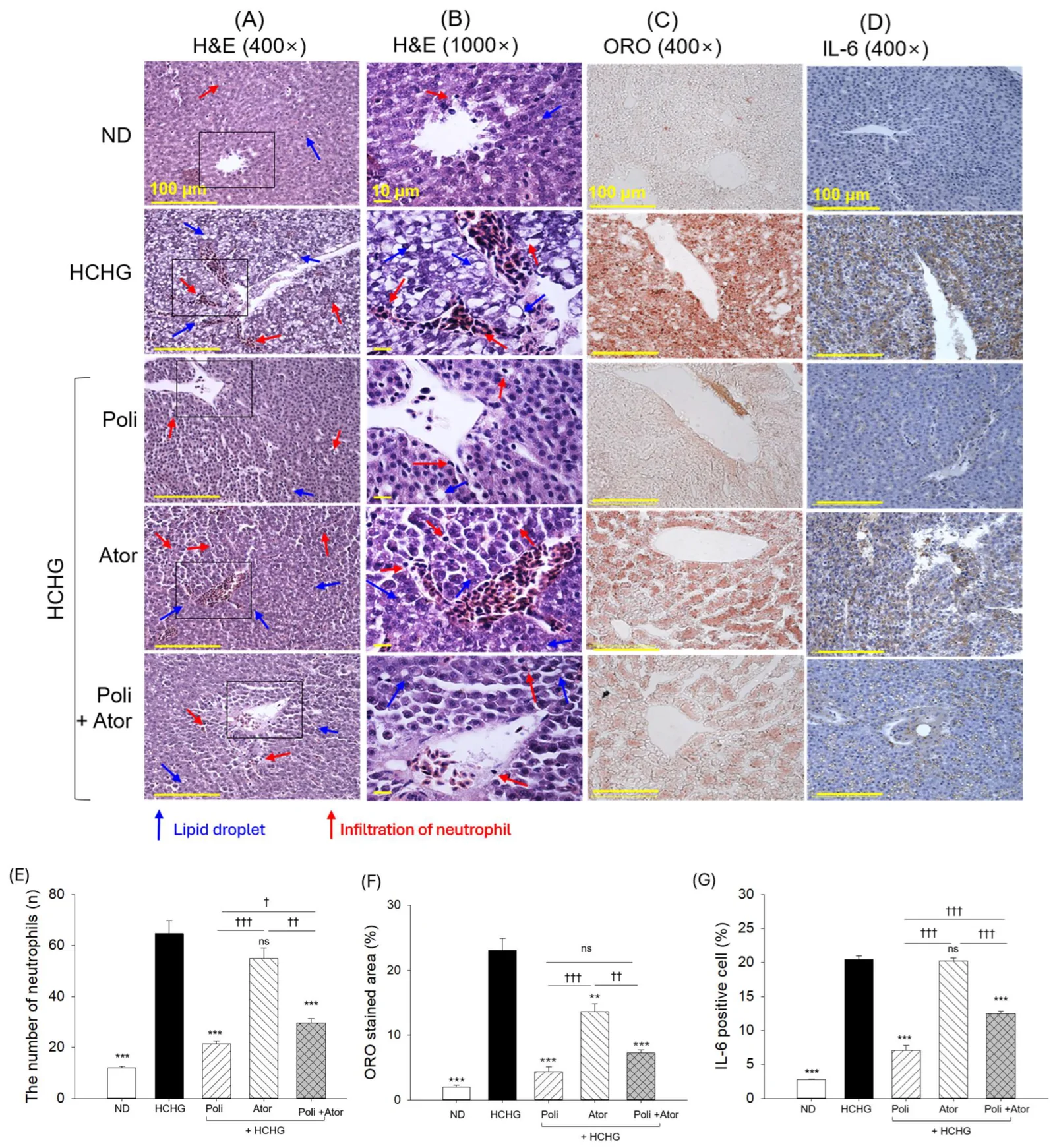
Liver histology of the zebrafish after 22 weeks of feeding on the different dietary formulations. (**A**) Hematoxylin and eosin (H&E) staining at 400× magnification. (**B**) H&E imaging of the section covered under the black box (shown at 1000× magnification). (**C**) Oil Red O (ORO) staining and (**D**) immunohistochemical (IHC) staining for the detection of interleukin (IL)-6. Quantification of (**E**) neutrophils, (**F**) ORO-stained area and (**G**) IL-6-stained area. Abbreviations: ND, normal diet; HCHG, high-cholesterol and high-galactose diet; Poli, policosanol; Ator: atorvastatin. ImageJ software (version 1.54s, https://imagej.net/ij; assessed in 7 April 2026) was used to quantify ORO and IL-6-stained area. For the quantitative analysis of neutrophils, ORO and IL-6-stained areas, three sections and five random fields per section were analyzed. ** and *** underscore the statistical difference at *p* < 0.05 and *p* < 0.001 between the groups relative to the HCHG group, while ^†^ (*p* < 0.05), ^††^ (*p* < 0.01) and ^†††^ (*p* < 0.001) depict the statistical difference between the marked groups evaluated by one-way ANOVA, followed by Tukey’s post hoc analysis; ns represents non-significant difference.

The H&E staining suggests the highest neutrophil counts in the HCHG group, which was notably 5.4-fold higher than the neutrophil counts observed in the hepatic tissue of the ND (control) group (Figure 6A,B,E). Compared with the HCHG group, neutrophil counts were reduced by approximately 3.3-fold and 2.2-fold in Poli and Poli+Ator-supplemented groups, respectively. Compared with the HCHG group, Ator supplementation did not significantly affect hepatic neutrophil counts.

The ORO staining revealed that HCHG-induced hepatic lipid accumulation was significantly reduced by Poli and Ator by approximately 5.3-fold and 1.7-fold, respectively (Figure 6C,F). In the Poli+Ator-supplemented group, a substantial reduction in lipid deposition was observed compared to the lipid deposition of the HCHG- and Ator-supplemented groups.

A significantly higher 7.6-fold IHC-stained area corresponding to the IL-6 production was observed in the hepatic section from the HCHG-supplemented group compared to the ND (control) group (Figure 6D,G). The supplementation with Poli and Poli+Ator effectively reduced HCHG-induced hepatic IL-6 production. No notable change in the hepatic IL-6 production was observed in the Ator-supplemented group compared to the HCHG group.

### 2.7. Hepatic Dihydroethidium (DHE) and Senescence-Associated β-Galactose (SA-β-Gal) Staining

**Figure 7:**
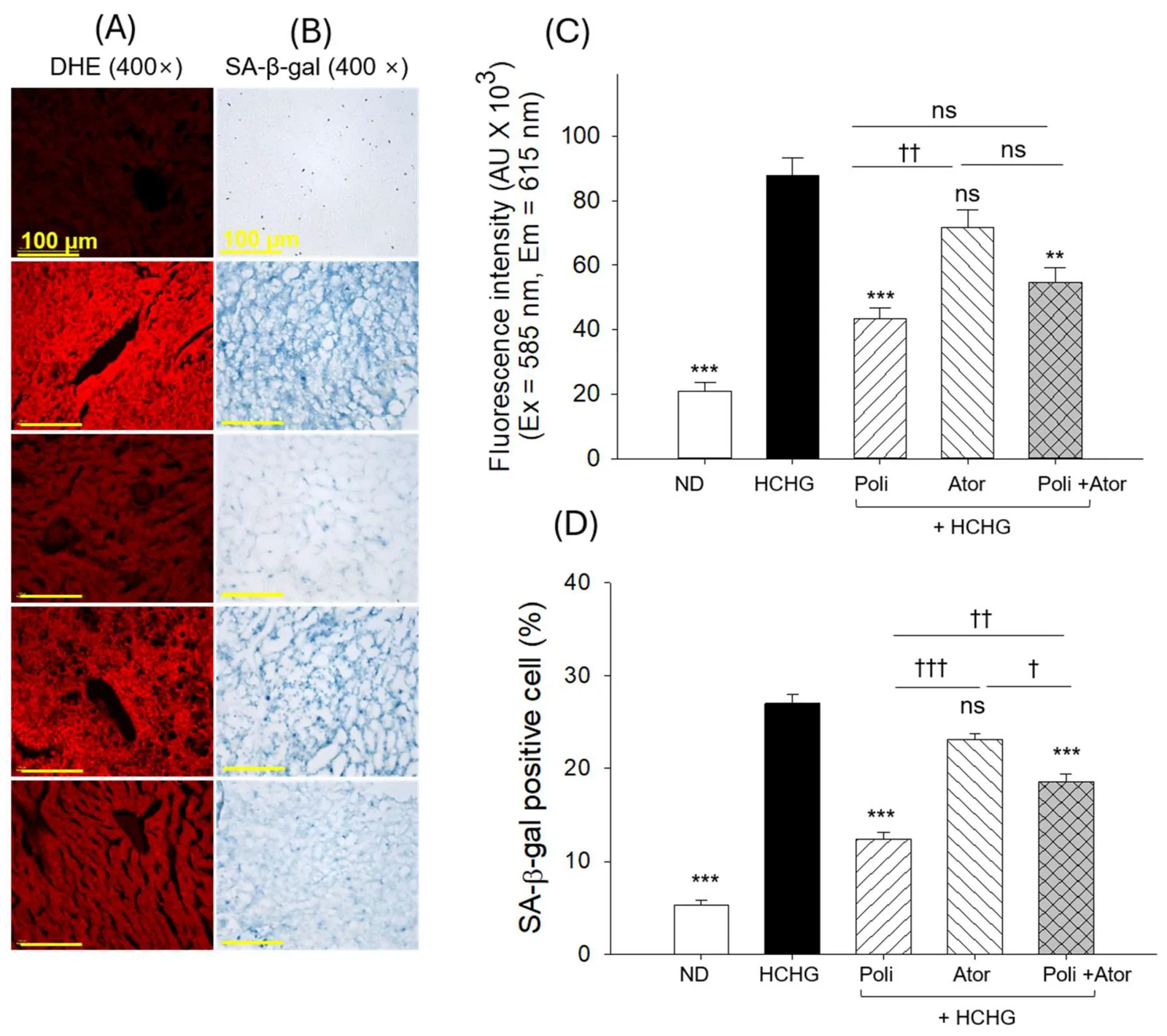
Dihydroethidium (DHE) staining (**A**) and senescent-associated β-galactosidase (SA-β-gal) staining (**B**) in the liver of zebrafish after 22 weeks of feeding the different dietary formulations. Quantification of the (**C**) DHE fluorescent intensity and (**D**) SA-β-gal-positive cells. Abbreviations: ND, normal diet; HCHG, high-cholesterol and high-galactose diet; Poli, policosanol; Ator: atorvastatin. ImageJ software (version 1.54s; https://imagej.net/ij; assessed in 7 April 2026) was used to quantify DHE- and SA-β-gal-positive cells. For the quantitative analysis, three sections and five random fields per section were analyzed. ** and *** underscore the statistical difference at *p* < 0.01 and *p* < 0.001, respectively, between the groups relative to the HCHG group, while ^†^ (*p* < 0.05), ^††^ (*p* < 0.01) and ^†††^ (*p* < 0.001) depict the statistical difference between the marked groups determined by one-way ANOVA, following Tukey’s post hoc analysis; ns indicates a non-significant difference between the groups.

The DHE fluorescence and SA-β-gal staining suggest a 4.2-fold and 5-fold elevation in oxidative stress and senescent-positive cells in the HCHG-supplemented group compared to that of the ND (control) group (Figure 7A,C). Supplementation with Poli and Poli+Ator significantly reduced hepatic oxidative stress and senescent-positive cells compared to the HCHG group (Figure 7A–D). No significant effect of Ator supplementation was observed on HCHG-elevated hepatic oxidative stress and senescent-positive cells.

### 2.8. Hepatic Function Enzymes

**Figure 8:**
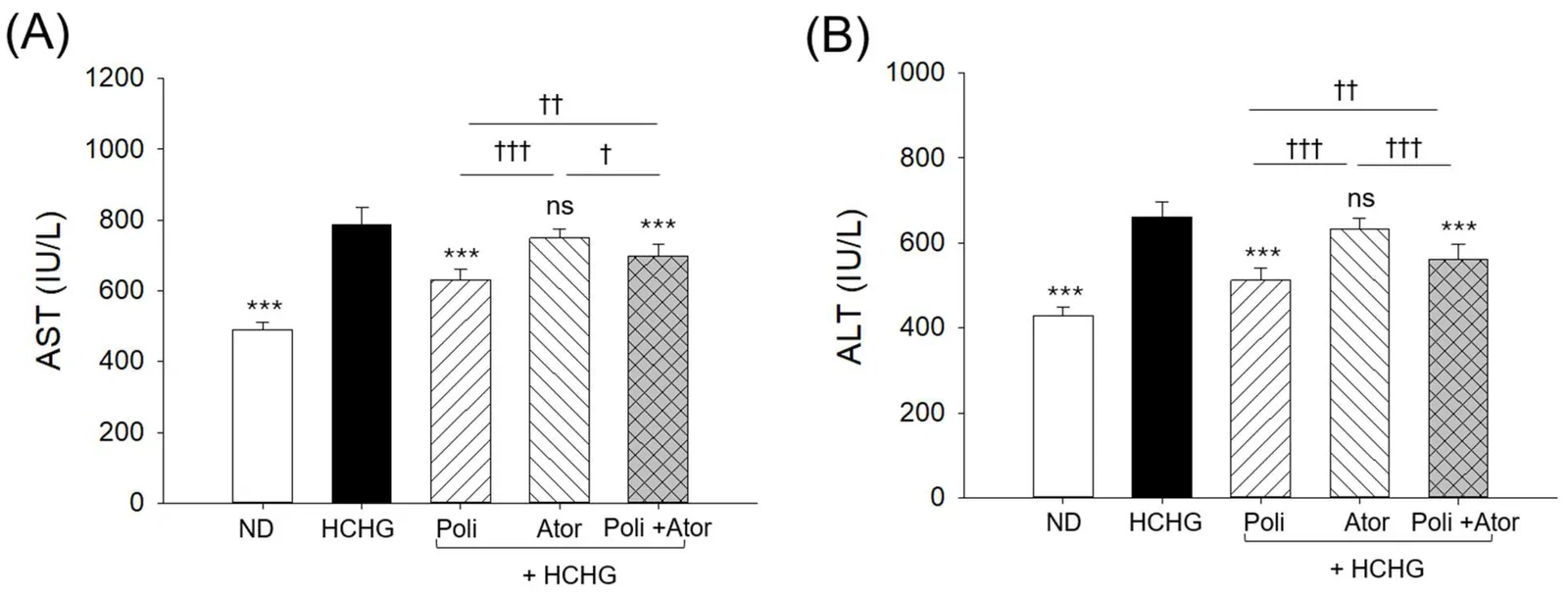
Blood levels of (**A**) aspartate aminotransferase (AST) and (**B**) alanine aminotransferase in zebrafish after 22 weeks of feeding the different formulated diets. Abbreviations: ND, normal diet; HCHG, high-cholesterol and high-galactose diet; Poli, policosanol; Ator: atorvastatin. Each point in the bar graph represents the mean ± SEM value obtained from three (*n* = 3) independent experiments. *** underscores the statistical difference at *p* < 0.001 between the groups relative to the HCHG group, whereas ^†^ (*p* < 0.05), ^††^ (*p* < 0.01) and ^†††^ (*p* < 0.001) depict the statistical difference between the marked groups determined by one-way ANOVA, following Tukey’s post hoc analysis; ns indicates a non-significant difference between the groups.

A significant 1.6-fold and 1.5-fold higher AST and ALT levels compared to the ND (control) group were observed in the HCHG-supplemented group (Figure 8A,B). The HCHG-induced elevation of AST and ALT levels was substantially reduced by supplementation with Poli and Poli+Ator. In contrast, no significant effect of Ator supplementation was observed in preventing the HCHG-induced elevation of AST and ALT levels.

### 2.9. Histological Analysis of Kidney

**Figure 9:**
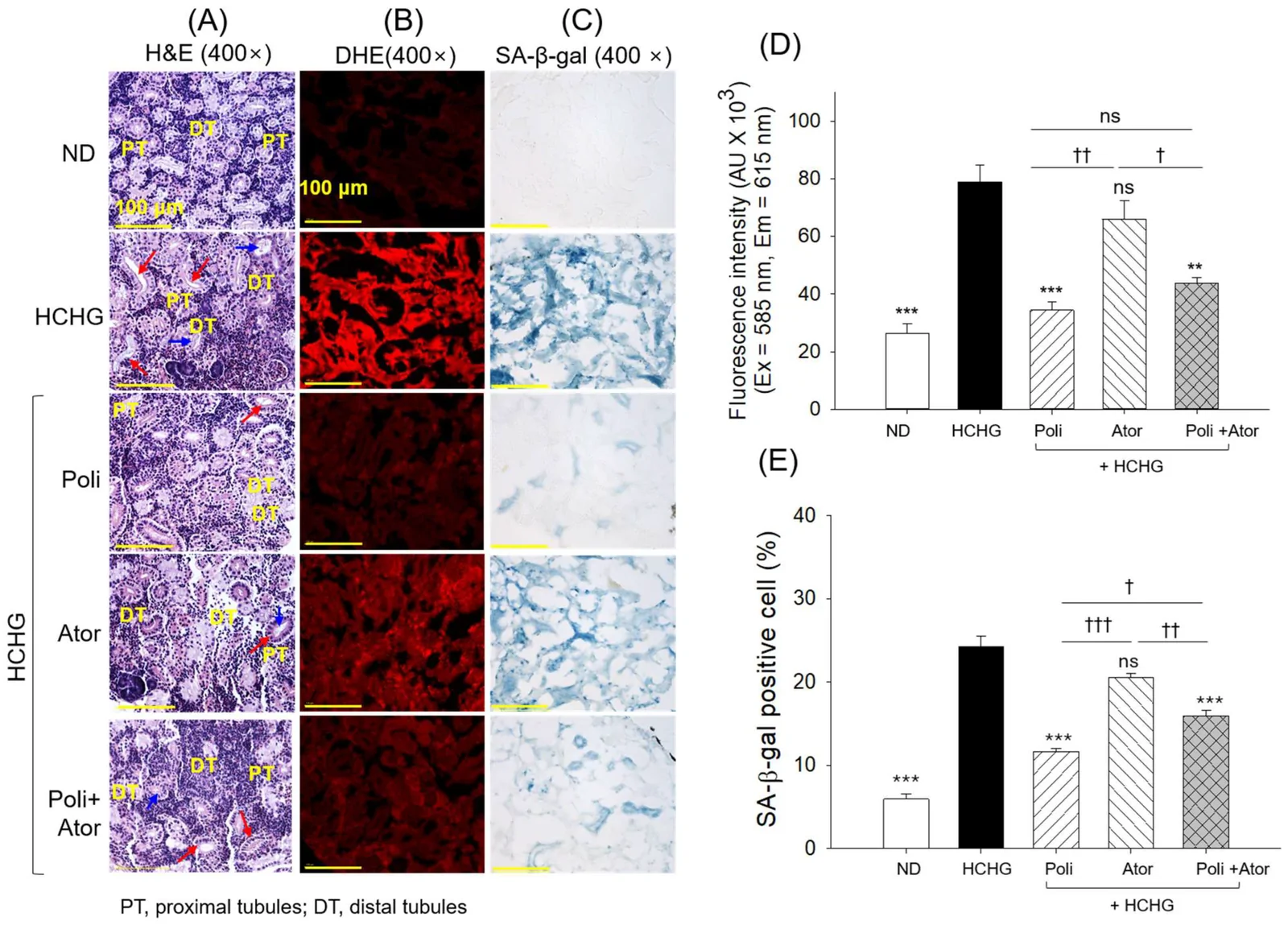
Kidney histology of the zebrafish after 22 weeks of feeding on the different dietary formulations. (**A**) Hematoxylin and eosin (H&E) stain at 400× magnification. Red color highlights the dilated tubular lumen while blue arrows indicate cellular debris in tubular lumen. (**B**) Dihydroethidium (DHE) fluorescent staining. (**C**) Senescence-associated β-galactosidase (SA-β-gal) staining. Quantification of (**D**) DHE fluorescent intensity and (**E**) SA-β-gal-positive cells. Abbreviations: ND, normal diet; HCHG, high-cholesterol and high-galactose diet; Poli, policosanol; Ator: atorvastatin. ImageJ software (version 1.54s; https://imagej.net/ij; assessed in 17 April 2026) was used to quantify DHE- and SA-β-gal-positive cells. For the quantitative analysis, three sections and five random fields per section were analyzed. ** and *** underscore the statistical difference at *p* < 0.05 and *p* < 0.001 between the groups relative to the HCHG group, whereas, ^†^ (*p* < 0.05), ^††^ (*p* < 0.01) and ^†††^ (*p* < 0.001) depict the statistical difference between the marked groups determined by one-way ANOVA, following Tukey’s post hoc analysis; ns indicates a non-significant difference between the groups.

The kidney histology revealed a well-structured and compact arrangement of proximal and distal tubules in the ND (control) group. A 22-week consumption of HCHG altered the proximal and distal tubular arrangement and slightly distorted the shape (Figure 9A). A notable elevation of the tubular lumen (indicated by red arrow) and cellular debris (indicated by blue arrow) in the tubular cast was observed in the kidney from the HCHG group. The consumption of Poli substantially prevented the adverse histological changes caused by HCHG; however, the rare presence of a dilated tubular lumen and cellular debris in the tubular cast was observed in some areas. In contrast, no protective effect of Ator supplementation was observed against HCHG-induced histological changes in the kidney; moreover, compared with HCHG, the Ator-supplemented groups showed greater tubular disarrangement, dilated tubular lumens, and cellular debris in the lumen. In contrast, Ator, in combination with Poli (i.e., Poli+Ator), showed a substantial protective effect against HCHG-induced histological changes in the kidney.

The DHE and SA-β-gal staining suggest the highest oxidative stress and senescent-positive cells in the kidney of the HCHG group, which were notably 2.9- and 4.1-fold higher than those observed in the ND (control) group (Figure 9B–E). A significant positive effect of Poli and Poli+Ator supplementation was observed in preventing HCHG-induced oxidative stress and senescent-positive cells. No significant effect of Ator was observed in preventing the generation of oxidative species and senescence induced by HCHG supplementation.

### 2.10. Histological Analysis of Intestine

**Figure 10:**
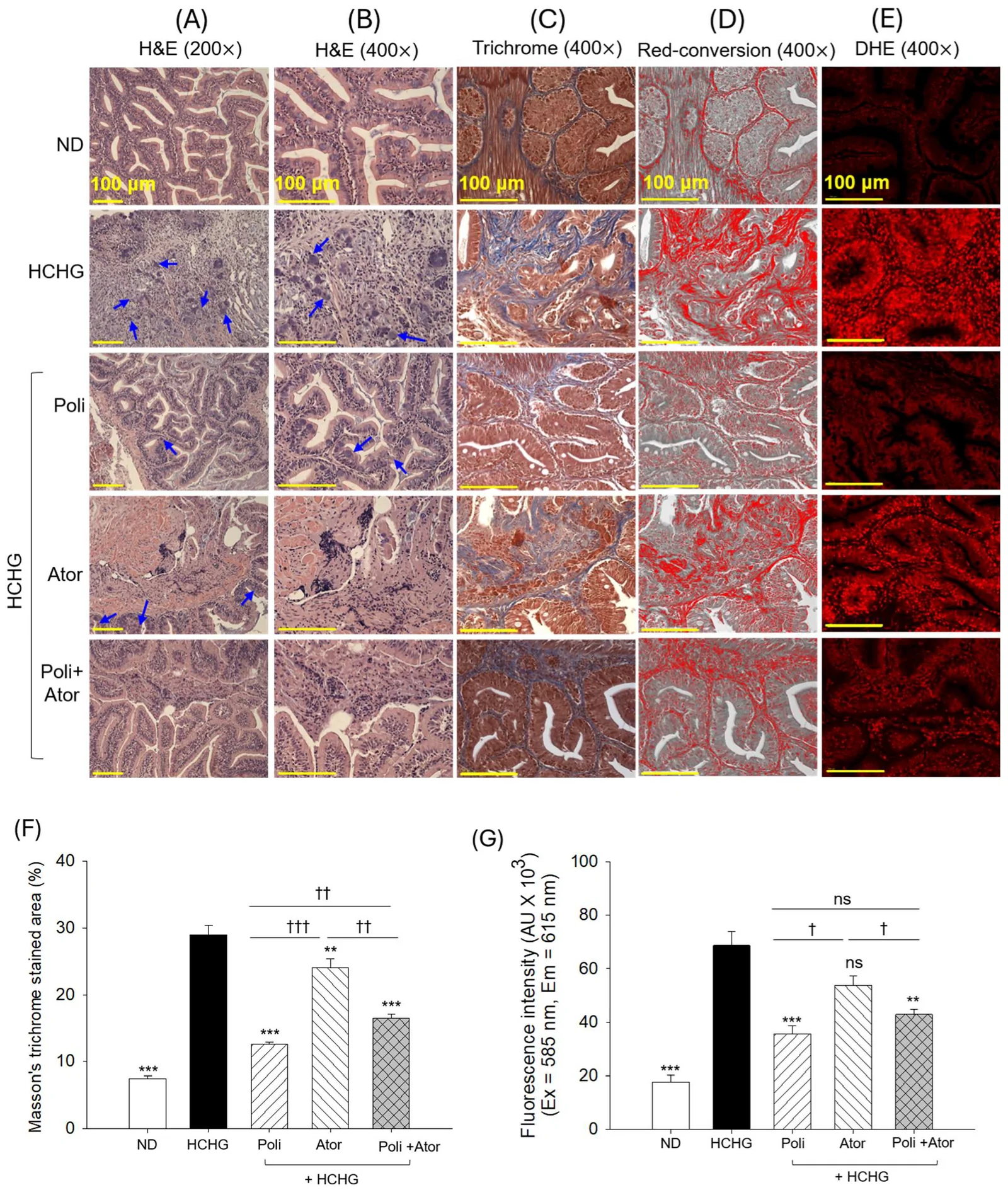
Histological analysis of the intestine from zebrafish after 22 weeks of feeding different formulated diets. Hematoxylin and eosin (H&E) staining at (**A**) 200× and (**B**) 400× magnification. (**C**) Masson’s trichrome-stained section at 400× magnification. Blue arrows highlight the shrinkage or swelling of the goblet cells. (**D**) Red-converted images of Masson’s trichrome-stained section using ImageJ software (https://image.net/ij, version 1.54s, assessed on 17 April 2026) at a blue color threshold value of 20–120. (**E**) Dihydroethidium (DHE) fluorescent staining. Quantification of (**F**) Masson’s trichrome-stained area and (**G**) DHE fluorescent intensity. Abbreviations: ND, normal diet; HCHG, high-cholesterol and high-galactose diet; Poli, policosanol; Ator: atorvastatin. ImageJ software was used to quantify collagen-stained areas and DHE fluorescence intensity. For the quantitative analysis, three sections and five random fields per section were analyzed. ** and *** underscore the statistical difference at *p* < 0.05 and *p* < 0.001 between the groups relative to the HCHG group, while ^†^ (*p* < 0.05), ^††^ (*p* < 0.01) and ^†††^ (*p* < 0.001) depict the statistical difference between the marked groups determined by one-way ANOVA, following Tukey’s post hoc analysis; ns indicates a non-significant difference between the groups.

The histological images obtained from the H&E staining revealed a well-defined villus structure and firmly attached lamina propria in the ND (control) group (Figure 10A,B). The consumption of HCHG substantially affected intestinal morphology, with disintegration of the villus structure and shrinkage or swelling of the goblet cells (indicated by the blue arrow). Supplementation with Poli and Poli+Ator substantially prevented HCHG-induced intestinal damage; however, enteric villus dissolution was observed in the intestinal tissue. No substantial protective effect of Ator supplementation was observed against HCHG-induced intestinal damage. Consistent with the H&E outcomes, the least collagenated area was detected in the ND (control) group, which was notably 3.8-fold lower than the collagenated area observed in the HCHG group (Figure 10C,D,F). Supplementation with Poli, Ator, and Poli+Ator significantly minimizes HCHG-induced collagenation, as reflected by collagenated areas that were 2.3-, 1.2-, and 1.8-fold lower, respectively, compared to the HCHG group. However, compared with the Ator-supplemented group, the Poli and Poli+Ator-supplemented groups showed 1.9- and 1.5-fold higher efficacy in mitigating HCHG-induced intestinal collagenation, respectively.

The DHE staining revealed a high level of oxidative species in the HCHG group, which was substantially reduced by 2.3-fold and 1.8-fold with supplementation with Poli and Poli+Ator, respectively (Figure 10E,G). In contrast, a non-significant effect of Ator was observed to attenuate HCHG-triggered oxidative species generation.

### 2.11. Histological Analysis of Testis and Ovary

**Figure 11:**
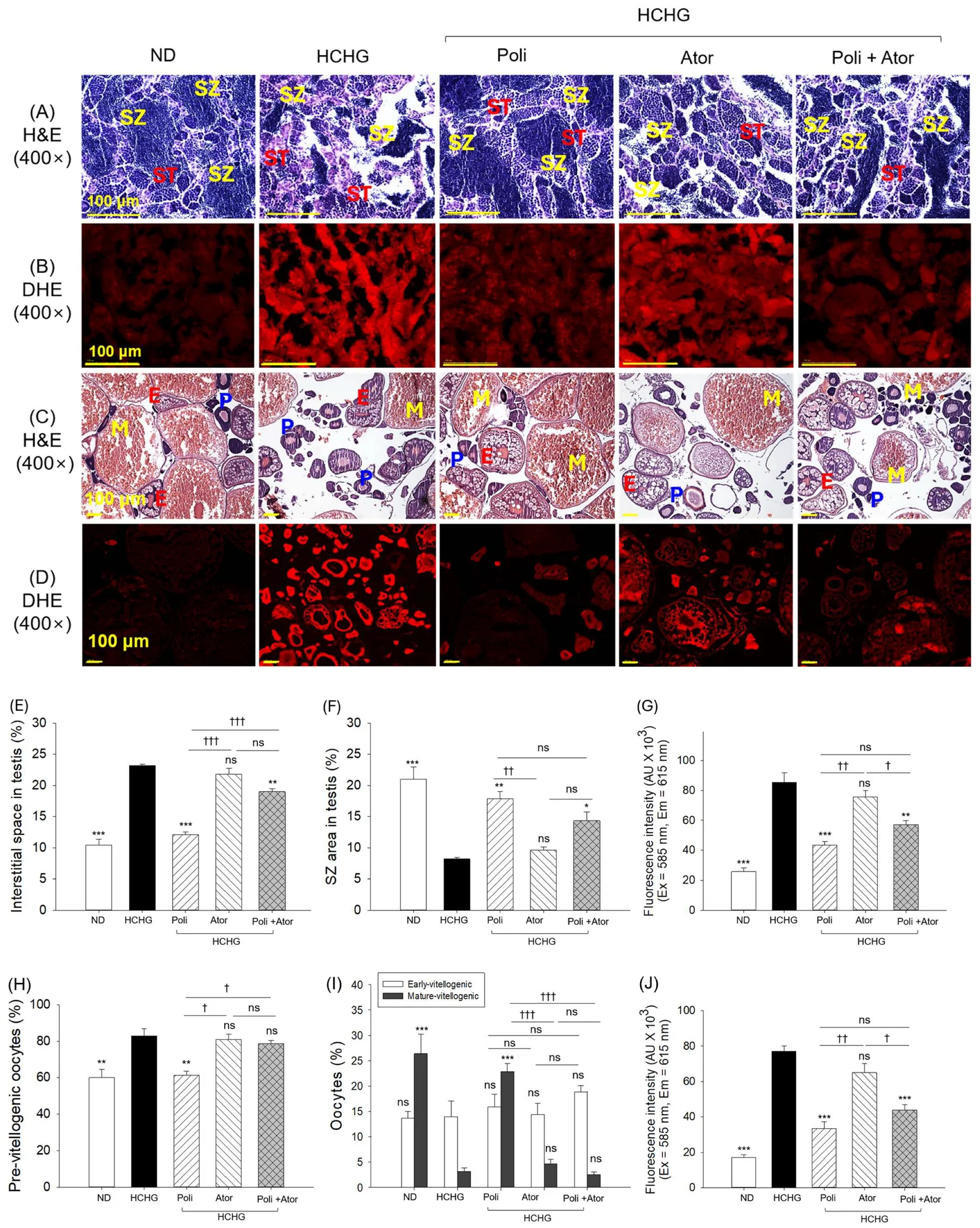
Histology of reproductive organs of the zebrafish after 22 weeks of feeding on the different dietary formulations. Testis (**A**) hematoxylin and eosin (H&E) staining and (**B**) dihydroethidium (DHE) fluorescent staining, at 400× magnification. ST and SZ highlight spermatocytes and spermatozoa, respectively. Ovary (**C**) hematoxylin and eosin (H&E) staining and (**D**) dihydroethidium (DHE) fluorescent staining, at 400× magnification. P, E and M represent pre, early and mature vitellogenic oocytes, respectively. Quantification of (**E**) interstitial space, (**F**) spermatozoa and (**G**) DHE fluorescent intensity in the testis section. Quantification of (**H**) pre-vitellogenic oocytes, (**I**) early and mature vitellogenic oocytes, and (**J**) DHE fluorescent intensity in the ovary section. Abbreviations: ND, normal diet; HCHG, high-cholesterol and high-galactose diet; Poli, policosanol; Ator: atorvastatin. For the quantitative analysis, three sections and five random fields per section were analyzed. ImageJ software (version 1.54s, https://imagej.net/ij; assessed in 17 April 2026) was used to quantify interstitial space, spermatozoa area and DHE fluorescent intensities. *, ** and *** underscore the statistical difference at *p* < 0.05, *p* < 0.01 and *p* < 0.001 between the groups relative to the HCHG group, while ^†^ (*p* < 0.05), ^††^ (*p* < 0.01) and ^†††^ (*p* < 0.001) depict the statistical difference between the marked groups determined by one-way ANOVA, following Tukey’s post hoc analysis; ns indicates a non-significant difference between the groups.

Supplementation of HCHG substantially impacted the testis, reflected by the lowest spermatozoa and highest interstitial space between the seminiferous tubules, which were significantly 2.6-fold lower and 2.2-fold higher than the respective areas observed in the ND (control) group. The supplementation of Poli and Poli+Ator effectively reduced the HCHG-elevated interstitial space by 1.9-fold and 1.2-fold, while enhancing the spermatozoa area by 2.2-fold and 1.8-fold, respectively. Similarly, the HCHG-induced elevation in oxidative species levels was significantly reduced by 1.9-fold and 1.4-fold in the Poli and Poli+Ator-supplemented groups (Figure 11B,G). No significant effect of Ator supplementation was observed in mitigating HCHG-elevated oxidative species generation, interstitial space, and reduced spermatozoa area.

The ovary histology revealed a notable 1.4-fold higher presence of pre-vitellogenic oocytes and 8.5-fold reduced mature oocytes in the HCHG-consuming groups than that of the ND (control) group (Figure 11C,H,I). Supplementation of Poli demonstrated a significant 1.4-fold reduction in pre-vitellogenic oocytes and a 7-fold increase in mature oocyte counts in the Poli-supplemented groups compared to the HCHG group. No significant effect of Ator and Poli+Ator was observed in mitigating HCHG-induced damage to ovarian cellular structure.

The DHE staining revealed a massive generation of oxidative species in the HCHG group, which was significantly reduced by 2.3-fold and 1.8-fold following intake of Poli and Poli+Ator (Figure 11D,J). No significant effect of Ator supplementation was observed in minimizing HCHG-provoked oxidative species generation.

### 2.12. Production and Development of Embryos

**Figure 12:**
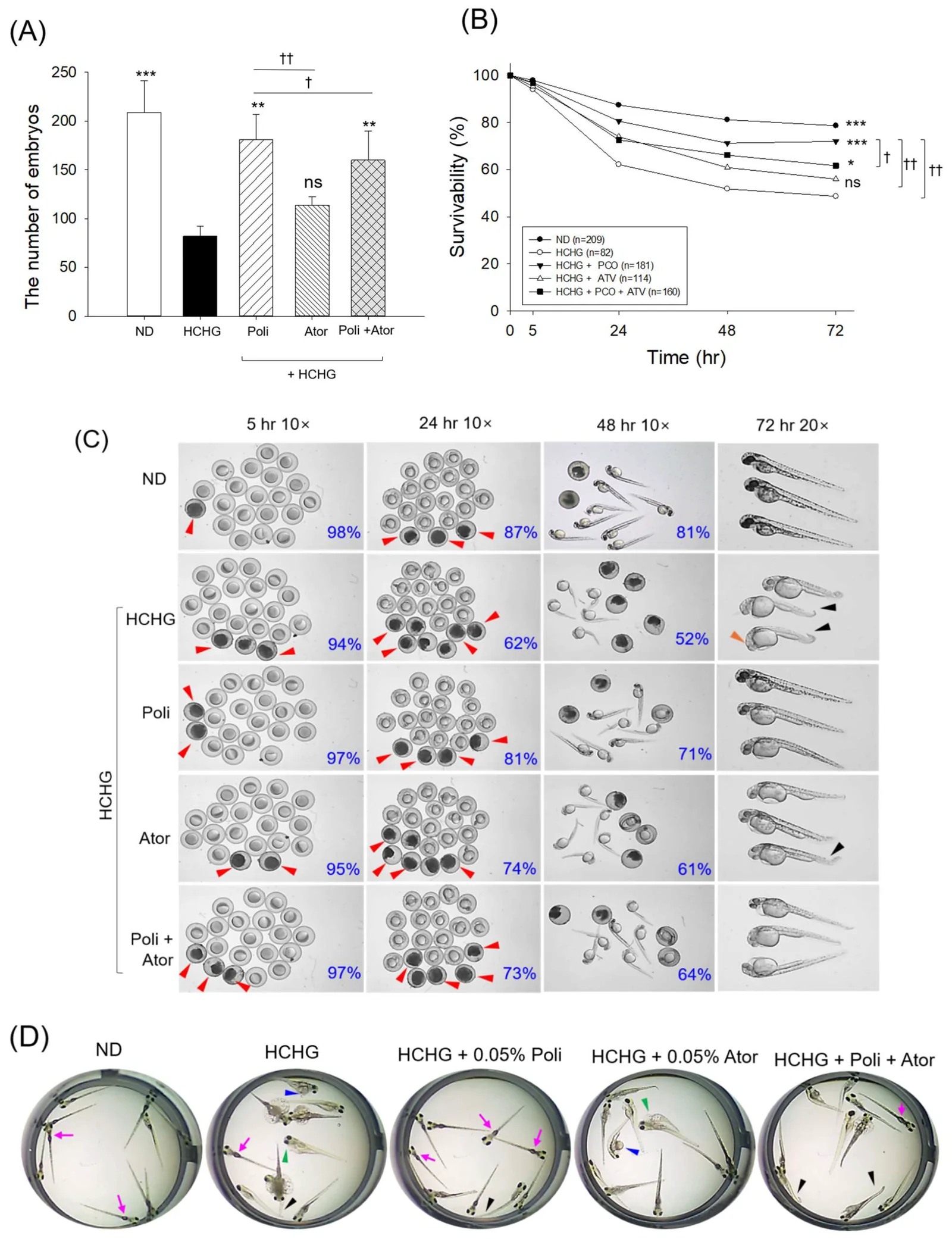
Egg-laying ability of zebrafish after 22 weeks of feeding on different dietary formulations. (**A**) Total number of produced embryos. (**B**) Embryo survival kinetics during 72 h post-fertilization. (**C**) Images depicting the morphology and development stage of embryos at 5 h, 24 h, 48 h and 72 h post-fertilization. Red and black arrows depict dead embryos and tail fin curvature. (**D**) Morphological images of the embryos at 144 h post-fertilization. Blue arrows highlight yolk sac edema, pink arrows highlight inflated swimming bladder, and green arrows highlight pericardial edema. Abbreviations: ND, normal diet; HCHG, high-cholesterol and high-galactose diet; Poli, policosanol; Ator: atorvastatin. Each point in the bar and line graphs represents the mean ± SEM value obtained from three (*n* = 3) independent experiments. *, ** and *** underscore the statistical difference at *p* < 0.05, *p* < 0.01 and *p* < 0.001 between the groups relative to the HCHG group, while ^†^ (*p* < 0.05), and ^††^ (*p* < 0.01) depict the statistical difference between the marked groups determined by one-way ANOVA, following Tukey’s post hoc analysis; ns indicates a non-significant difference between the groups.

Compared to average embryos produced in the ND (control) group (209), severely compromised embryo production (82) was noticed in the HCHG-consuming zebrafish (Figure 12A). The HCHG-compromised embryo production ability was significantly elevated by 2.2-fold and 1.9-fold in the Poli and Poli+Ator-supplemented groups. A non-significant effect of Ator supplementation was noticed on the HCHG-diminished embryo production ability.

Furthermore, the embryo survival kinetics, as depicted in Figure 12B,C, suggest the lowest embryo survival in the HCHG groups. In contrast to this, the embryos produced in the Poli and Poli+Ator-supplemented groups showed improved embryo survival, which was significantly 1.5-fold and 1.3-fold higher, respectively, than the embryo survival in the HCHG group at 72 h post-fertilization. However, the embryo survival from the Ator-supplemented group remained statistically similar to the survival of embryos observed in the HCHG-supplemented group.

Morphological analysis at 72 h and 144 h post-fertilization revealed that ~15% of the embryos from the HCHG groups showed some degree of developmental deformities concerning tail fin curvature, pericardial edema, and yolk sac edema (Figure 12C,D). In contrast, embryos from the Poli and Poli+Ator groups showed normal embryonic morphology; however, ~5% of embryos exhibited minor developmental deformities, primarily involving tail fin curvature. Unlike this, ~11% of embryos in the Ator group showed developmental deformities related to pericardial edema and yolk sac edema.

## 3. Discussion

High-cholesterol and high-galactose consumption led to several adverse effects and cause damage to a variety of organs. HCHG intake induces a variety of metabolic changes and is considered an appropriate dietary model for inducing metabolic stress that resembles the pathophysiology of human disease [38]. In the present study, a 22-week intake of HCHG, in the presence of Poli, Ator, and Poli+Ator, was evaluated to determine their efficacy in mitigating HCHG-induced metabolic changes and organ damage.

Ator is the commonly used statin to treat dyslipidemia. Ator is a competitive inhibitor of HMG-CoA reductase, a rate-limiting enzyme in cholesterol biosynthesis, thereby regulating cholesterol levels [5]. Likewise, Poli has been documented to regulate HMG-CoA reductase; consequently, it regulates cholesterol levels [22]. In addition, Poli improved cholesterol efflux capacity [24] and had a substantial effect on cholesterol catabolism in the liver by converting cholesterol into bile acid [39,40], with subsequent fecal excretion contributing substantially to the regulation of cholesterol levels. In line with the earlier findings, Ator and Poli significantly improved the HCHG-triggered dyslipidemia. Nevertheless, compared with individual supplementation (Poli and Ator), the combination of Poli+Ator exhibited a significantly greater effect in decreasing the elevation of HCHG-induced TC, TG, and LDL-C levels. Both Poli and Ator supplementation substantially improved HDL-C levels; however, Poli+Ator supplementation was the most effective, yielding the largest increases in HDL-C and the HDL-C/TC ratio. The findings are consistent with our earlier report, in which supplementation with Poli+Ator at the higher dose (0.1% *w*/*w*) for a short duration (12 weeks) effectively improved the HCHG-disturbed HDL-C/TC ratio in zebrafish. Taken together, the outcomes from both studies further support the effectiveness of Poli+Ator in improving the HDL-C/TC ratio. Poli’s impact in inhibiting the cholesteryl ester transfer protein (CETP) activity [41] and upregulating the expression of apolipoprotein A-I (apoA-I), a major HDL protein [24], are the key events to augment the HCHG-diminished HDL-C levels. The outcomes of the current findings align with earlier published reports in which the comparative effects of Poli and Ator were tested in human participants [29,30]. Consistent with the current findings, both Poli and Ator reduced elevated TC, LDL-C, and TG levels after 8 weeks of supplementation in human participants with dyslipidemia and type 2 diabetes [29] and with type 2 hypercholesterolemia [30]. Consistent with the present findings, Ator supplementation in humans showed a greater reduction in TC and LDL-C levels than the Poli-supplemented participants [30]. In support of the current finding, a notable increase in HDL-C level was observed in type 2 hypercholesterolemia individuals following the Poli supplementation [30]. However, unlike the present outcomes, no effect of Ator on HDL-C levels was observed in human participants [30]. Also, contrary to the present findings, co-supplementation of Poli with Ator for 12 weeks did not significantly enhance the lipid-lowering effect of Ator in human subjects [28].

Excessive HCHG consumption leads to oxidative stress, as evidenced by elevated blood MDA levels and reduced sulfhydryl content, both of which are common oxidative stress markers associated with various pathological conditions [42,43,44]. Consistently, a diminished blood FRA and PON activity in response to HCHG indicates a compromised antioxidant and oxidative stress environment. Supplementation with Poli effectively counteracts HCHG-induced increases in oxidative stress and impairments in antioxidant activity. The findings are in good agreement with earlier reports documenting Poli’s inhibitory effect on MDA [45] and the augmentation of sulfhydryl content, PON, and FRA activities [46], resulting in a beneficial effect against oxidative stress. Reports have shown that Ator can induce oxidative stress in blood and organs, as indicated by elevated MDA levels and diminished glutathione content [47]. However, no such adverse effects were observed by us in the Ator-supplemented group during a 22-week feeding study. Although Ator alone did not mitigate HCHG-induced oxidative stress, its combination with Poli markedly suppressed HCHG-induced oxidative stress and improved the antioxidant defense system. The findings are consistent with an earlier report [31], in which a 12-week intake of Poli+Ator at 0.1% produced similar outcomes, supporting the beneficial effect of this combination in restoring the disturbed oxidative stress markers induced by HCHG intake.

A high-fat diet, high galactose intake, and oxidative stress cause insulin resistance and negatively affect insulin secretion, leading to hyperglycemia [48,49]. Studies documented the adverse effect of statins (including Ator) on type 2 diabetes [17,18,19]. Even more, in diabetic rats, Ator has been documented to cause insulin resistance, suggesting its adverse effect on glycemic regulation [50]. In contrast, the role of Poli in insulin secretion and sensitization mediated by the AMPK and PI3K/AKT signaling pathways has been described as a mechanism of glycemic control [26]. Also, the inhibitory effect of Poli on CETP activity has been documented [41], which may contribute to lower blood glucose levels. This notion is in accordance with previous report documenting the impact of CETP inhibitors on reducing blood glucose levels [51]. Herein, consistent with the literature, a reduction in blood glucose was observed in the Poli and Poli+Ator-supplemented groups, whereas no blood glucose-lowering effect was observed in the only Ator-supplemented group. Consistent with this study’s findings, Poli intake was effective in reducing blood glucose levels in human patients with type 2 hypercholesterolemia, whereas Ator failed to reduce blood glucose levels [30]. The current results clearly demonstrate that the combination of Poli and Ator substantially reduced the HCHG-elevated glucose levels in zebrafish. Due to variation in certain physiological aspects [37] and in human lifestyle and behavior [52], the results obtained from the zebrafish model need to be evaluated critically in the context of its therapeutic application in humans.

A notably high recovery of HCHG-impaired amputated tail fin regeneration was observed in the Poli-supplemented group, while no beneficial effect was noted for the Ator-consuming zebrafish. However, Ator in combination with Poli (Poli+Ator) substantially promoted the amputated tail fin regeneration. A substantially low glucose level in the Poli and Poli+Ator groups might be the reason for the high tail fin regeneration in these groups. The notion is consistent with the fact that high glucose levels cause tissue inflammation and altered vascular endothelium growth factor signaling, consequently interfering with tissue regeneration and wound healing [53]. Nevertheless, a detailed molecular mechanistic study is required to establish any confirmatory evidence for the tissue regeneration ability.

High-cholesterol induces fatty liver [54], while high-galactose consumption leads to oxidative stress and inflammation [55]. Herein, the HCHG-induced liver damage is substantially protected by the Poli and Poli+Ator. However, no notable effect was observed for the Ator supplementation. The positive effect of the Poli on fatty liver, inflammation, and oxidative stress has been described [56], supporting the present findings. Several published reports documented Ator hepatotoxic effects accompanied by oxidative stress [57]. However, in the present study, we did not observe any adverse effect of Ator on the aggravation of liver damage caused by HCHG, and instead observed no response. Consistent with the histological findings, the HCHG group exhibited elevated blood AST and ALT levels, two established biomarkers of hepatic injury and liver dysfunction [58]. These enzyme levels remained statistically unchanged in the Ator-supplemented group, whereas significant reductions were observed in the Poli- and Poli+Ator-supplemented groups, further supporting their hepatoprotective effects. The present results corroborated earlier studies demonstrating the positive role of Poli in reducing AST levels in type 2 hypercholesterolemia patients [30], whereas Ator exhibited no effect. Even more Atro has been reported to elevate the ALT level in patients with dyslipidemia and type 2 diabetes mellitus [29], underscoring its negative implications for liver health.

High galactose consumption has been recognized to damage the kidney [59], through the induction of oxidative stress and accumulation of AGEs [60]. In the present study, Poli effectively mitigates kidney damage by suppressing oxidative species production. In addition, Poli has been documented to prevent glycation [23], which is an important step in attenuating AGE formation and protecting the kidneys. An adverse effect of oxidative stress and AGEs has been documented for kidney damage [60]; Poli’s effect to inhibit oxidative stress and AGEs formation [23] leads to protective events against HCHG-induced kidney damage. Herein, no effect of Ator supplementation was noticed to protect against kidney damage; however, in response to Poli+Ator, it substantially mitigates kidney damage. The lower senescence in response to Poli and Poli+Ator supplementation can be justified by the lowered oxidative species levels in these groups. As oxidative stress has been recognized as a key contributor to cellular senescence [61,62]. Kidney histological outcomes are consistent with blood sulfhydryl content findings, with substantially lower sulfhydryl content in the HCHG group, which was substantially elevated by supplementation with Poli and Poli+Ator. Notably, a low sulfhydryl content has been recognized as a marker of poor kidney health and disease [63].

High-cholesterol and galactose damage reproductive organs [64]. Herein, in response to HCHG, damage to the testis and ovary was observed, which was substantially protected by the supplementation of Poli and Poli+Ator. Studies have also documented the adverse effect of statins like Ator on testis and reproductive organs [13]. However, in the present 22-week consumption study, we did not observe any aggravating effect of Ator on HCHG-impaired reproductive health, although Ator showed a non-protective effect against HCHG-induced damage. Lower production of oxidative species in the Poli and Poli+Ator-supplemented groups reflects their antioxidant properties, which contribute to the protection of the testis and ovaries, as the protective effects of antioxidants have been well established for the ovary [65] and testis [66]. The current findings are corroborated by earlier reports [31] documenting that intake of Poli and Poli+Ator has a substantial beneficial effect on zebrafish reproductive organs and embryo production ability.

Limitations and prospective studies: This study was conducted with a single concentration of Poli and Atro combination; thus, it does not indicate whether the interaction between the two is synergistic or additive. Future studies will be conducted with different dose combinations of Poli and Ator to determine the dose–response curve and establish whether the interaction between these two is synergistic, additive, or independent. Furthermore, this study demonstrates the beneficial effects of Poli and Poli+Ator on oxidative variables, glucose levels, inflammation, and cellular senescence; however, the mechanistic aspects of their effects on genes and proteins at the molecular level were not investigated, which remains another limitation of the present study. Future studies will be conducted to address this limitation to explore the molecular events mediated by the Poli and Poli+Ator. Although zebrafish share several important physiological features with humans, there are some limitations also; for instance, zebrafish are poikilothermic (cold-blooded) unlike homeothermic (warm-blooded) humans [37]. Consequently, zebrafish metabolic rate is more strongly influenced by environmental conditions [67]. In addition, zebrafish exhibit differences in lipoprotein profiles compared to humans, including a higher proportion of apolipoproteins (~36% vs. 10%) and a higher HDL-C/TC ratio (~70% vs. 25%) [68]. Also, zebrafish LDL have higher triglycerides and lower cholesterol content [68]. Therefore, the findings from zebrafish should be validated in other models and in humans before concluding their applicability in humans.

## 4. Materials and Methods

### 4.1. Materials

Atorvastatin (PHR1422-1G) was purchased from Sigma-Aldrich (St. Louis, MO, USA), while policosanol (batch#310030324), extracted from Cuban sugarcane, was sourced from the National Centre for Scientific Research (CNIC), Havana, Cuba and was provided by Raydel Pty Ltd., Thornleigh, NSW, Australia. A detailed composition and certificate of policosanol analysis is provided as Supplementary Table S1. All the other chemicals and reagents were of analytical grade and used as supplied unless otherwise stated.

### 4.2. Zebrafish Maintenance

Adult zebrafish (*Danio rerio*, wild-type AB strain, 16 weeks old) of mixed sex were housed in a glass water tank equipped with a constant water supply maintained at 28 °C under the influence of 14 h light and 10 h dark alternative photoperiods. The water for zebrafish culturing has DO 8 mg/L, pH 7.3, chlorine 0.18 mg/L and turbidity 16 NTU, with total bacterial counts < 100 cfu without the presence of any pathogens. Water quality analysis was conducted by Kirim Life Science Co., Ltd. (Daegu, Republic of Korea), and the water quality analysis certificate is provided in Supplementary Table S2. Furthermore, the water was filtered sequentially through a 5 μm microdepth filter, activated carbon, and a 1 μm microdepth filter, and was treated with UV prior to being supplied to the zebrafish. Zebrafish were strictly maintained on the standard guidelines of Animal Care and Use [69] as recommended by the Raydel Research Institute (RRI-24-001, date of approval 2 September 2024). Fish were fed twice a day (9 am and 6 pm) with a commercial normal diet (ND) for zebrafish (Tetrabit GmBH, D49307, Melle, Germany). Zebrafish were kept under these conditions for 1 week to acclimatize to the environment before the main experiment.

### 4.3. Preparation of Different Diets

The ND was used as a base material to prepare four distinct formulated foods. ND was mixed with 4% (*w*/*w*) cholesterol and 30% (*w*/*w*) galactose to prepare the high-cholesterol and high-galactose diet (HCHG). The HCHG was supplemented with 0.05% (*w*/*w*) policosanol (Poli), 0.05% (*w*/*w*) atorvastatin (Ator), or 0.05% (*w*/*w*) each of policosanol and atorvastatin to prepare three distinct HCHG-enriched diets, named HCHG+Poli, HCHG+Ator, and HCHG+Poli+Ator. For preparing the HCHG diet, 132 g of ND was supplemented with 8 g of cholesterol and thoroughly mixed with a spatula. Subsequently, chloroform (~100 mL) was added to the mixture (ND+cholesterol), and the mixture was mechanically agitated to ensure proper distribution of cholesterol. Later, the chloroform was completely evaporated in a fume hood to obtain the high-cholesterol (HC) diet. The prepared HC diet was supplemented with 60 g of galactose, properly mixed with a spatula, and then distilled water (~300 mL) was added. The mixture was blended properly with a spatula and left for 4 h at room temperature (RT). Finally, the mixture was re-agitated (~10 min) and processed for freeze-drying to evaporate water. The freeze-dried sample was named the HCHG diet containing 4% (*w*/*w*) cholesterol and 30% (*w*/*w*) galactose.

For the preparation of HCHG enriched with Poli and Ator, 131.9 g of ND was supplemented with 8 g of cholesterol and mixed thoroughly using a spatula. Subsequently, chloroform (~100 mL) was added to the mixture, and the mixture was mechanically agitated to ensure proper distribution of cholesterol. The chloroform was then completely evaporated in a fume hood to obtain the HC diet. The prepared HC diet was subsequently supplemented with 60 g of galactose and 0.1 g of Poli or Ator, then mixed thoroughly with a spatula, followed by the addition of distilled water (~300 mL). The mixture was blended properly with a spatula and allowed to stand at RT for 4 h. Finally, the mixture was re-agitated (~10 min) and subjected to freeze-drying to evaporate water. The freeze-dried sample was designated as HCHG enriched with Poli or Ator and named as HCHG+Poli and HCHG+Ator. Similarly, the HCHG diet was supplemented with 0.1 g each of Poli and Ator to prepare the HCHG-enriched Poli and Ator diet, abbreviated as HCHG+Poli+Ator. The prepared diets, HCHG and HCHG+Poli, HCHG+Ator and HCHG+Poli+Ator, were stored in a refrigerator (4 °C) for further use.

The 0.05% dose was determined based on a preliminary study in which we fed Ator to hyperlipidemic-hyperglycemic zebrafish at concentrations of 0.0, 0.01, 0.025, 0.05, 0.75, and 1.0% under the influence of an HCHG diet. After 4 weeks of feeding, total blood cholesterol (TC) and triglyceride (TG) levels were measured. Both TC and TG levels decreased substantially with increasing Ator concentration from 0.01 to 0.05%; however, further increases in Ator concentration produced no significant reduction in TC and TG levels. Therefore, 0.05% Ator was selected as the lowest concentration that produced a substantial reduction in blood TC and TG levels and was used for subsequent experiments. For comparative analysis, policosanol was also used at the same concentration (0.05%).

Before starting the main feeding experiment, the liking and disliking of zebrafish toward ND, HCHG, HCHG+Poli, HCHG+Ator, and HCHG+Poli+Ator formulated diets was assessed by the food consumption test using the formula: [total amount of the given food (mg) − remaining amount of food (mg)/Total amount of the given food (mg)] × 100. Across all groups, 95–100% of food was consumed within 15 min of dietary exposure, suggesting that zebrafish have a similar preference for both formulated diets.

### 4.4. Feeding Zebrafish with Different Diets, Body Weight and Survival

**Figure 13:**
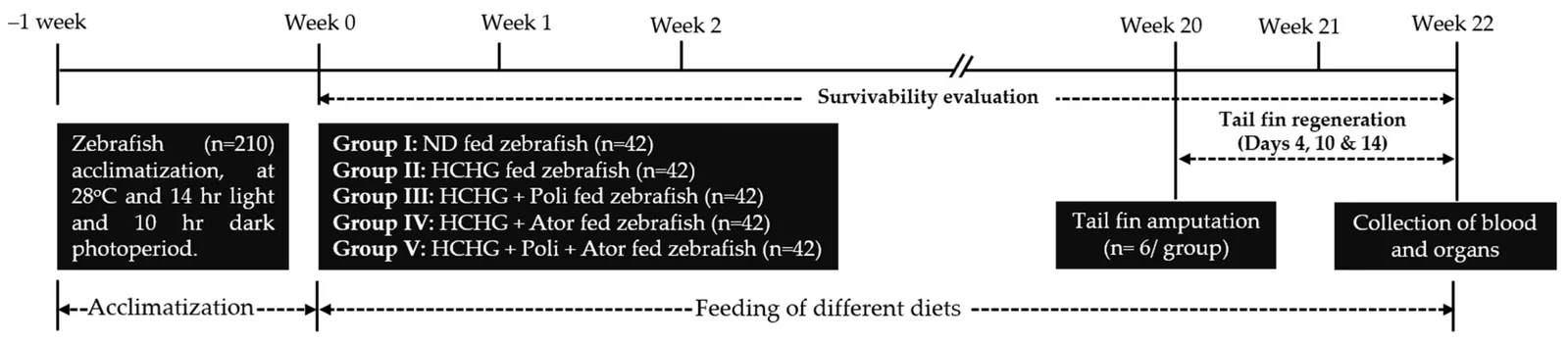
Study layout for zebrafish distribution across different groups and feeding till week 22. ND: normal diet; HCHG: high-cholesterol and high-galactose diet; Poli: policosanol; Ator: atorvastatin.

Initially, 210 adult zebrafish (105 males and 105 females) were maintained in two separate tanks according to sex. They were then randomly allocated into five experimental groups (*n* = 42/group), with each group subdivided into three independent tanks (*n* = 14/tank), containing an equal sex distribution of 7 males and 7 females. Therefore, for a particular group, three replicates were made (3 tank × 14 fish), of which half of the fish were male, and half were female. Zebrafish in group I was fed with an ND diet, while zebrafish in group II were fed with an HCHG diet. Zebrafish in groups III, IV and V were fed with HCHG+Poli, HCHG+Ator and HCHG+Poli+Ator diets (Figure 13). Zebrafish in the respective groups were fed a specialized diet for 22 weeks, including ND, HCHG, HCHG+Poli, HCHG+Ator, and HCHG+Poli+Ator. A specified diet (10 mg/zebrafish) was provided twice (at 9 am and 6 pm), equivalent to a cumulative diet of 200 mg/tank/day. As each tank contained 14 fish, it received 140 mg of diet (10 mg/fish × 14 fish) in the morning (9 am), and a similar amount was provided in the evening (6 pm). Consequently, each tank received a total of 280 mg of diet/day (equivalent to 20 mg of diet/fish/day).

Body weight of the zebrafish in different groups was measured at the beginning (week 0) and after the final 22 weeks of feeding. Survival rate of zebrafish in each group was assessed daily until week 22 of feeding.

### 4.5. Amputation of Tail Fin

Post 20 weeks of feeding, six zebrafish from each group (two from the three distinct tanks) were randomly selected and submerged in 0.1% of 2 phenoxyethanol for 2 min to anesthetize. The anesthetized zebrafish tail fin was surgically amputated near the dermal rays and immediately photographed under the stereo microscope. The amputated tail was further visualized on day 4, day 10 and finally on day 14 for the tail fin regeneration and imaged under the microscope. All images from the different groups were processed to quantify the regenerated tail fin area using ImageJ (https://image.net/ij, version 1.54s, assessed in 17 April 2026).

### 4.6. Collection of Blood and Organs

For the collection of blood and organs (after 22 weeks of feeding), zebrafish were euthanized using hypothermic shock. Blood from the zebrafish (~3 to 5 μL/fish) was immediately collected and mixed with 1 mM EDTA solution in phosphate-buffered saline (PBS) in a 2:3 ratio. Notably, the blood from zebrafish of each tank (as mentioned in Section 4.4) was pooled in a single tube and processed for centrifugation (6000 rpm, 10 min). The supernatant (plasma) was collected and preserved in the refrigerator (4 °C) for further analysis.

Different organs (liver, kidney, intestine, testis and ovary) were dissected under a microscope and immediately stored in 10% formalin for subsequent histological analysis. Notably, the specified organ from zebrafish of each tank (as mentioned in Section 4.4) was pooled in a single tube.

### 4.7. Analysis of the Blood

The plasma was processed to evaluate blood lipoprotein and hepatic function biomarkers by quantifying total cholesterol (TC), triglycerides (TG), high-density lipoprotein cholesterol (HDL-C), aspartate aminotransferase (AST), and alanine aminotransferase (ALT) using commercial assay kits, following the manufacturer’s recommended methodology. The diagnostic kits for quantifying TC (AM 202-K), TG (AM 157-K), HDL-C (AM 203-K), AST (AM 102-K), and ALT (AM 103-K) were procured from Asan Pharmaceutical in Hwasung, Republic of Korea. A detailed methodology for the quantification of TC, TG, LDL-C, HDL-C, AST, and ALT is provided in Supplementary Method Section S1.

Plasma malondialdehyde (MDA), sulfhydryl content, ferric ion reduction ability (FRA), and paraoxonase (PON) activity were determined using the previously described method [70]. A detailed methodology is provided in Supplementary Method Section S2.

### 4.8. Histological Analysis

For histological analysis, different organs were individually embedded in the Surgipath FSC22 frozen-section solution (3001480, lot no. 072325, Leica, Nussloch, Germany). The sample tissue was placed in the center of the object holder, and FSC22 frozen-section solution was added to completely cover the tissue. The object holder (with embedded sample) was then placed in the liquid nitrogen chamber to allow the solution to solidify (5 min). The solid block containing tissue was then stored in a deep freezer (−21 °C) for 24 h to stabilize it. Finally, the solidified tissue was sectioned (7 μm-thick slices) using a cryo-microtome (Leica CM-1510S, Nussloch, Germany).

The tissue section (7 μm thick) was processed for examination of histological changes using Hematoxylin and eosin (H&E) staining, following the previously described method [71]. In brief, the tissue section (7 μm thick) was covered with Ventana HE 600 hematoxylin solution (lot no. N11615, Roche, Tucson, AZ, USA) for 5 min, followed by 1 min of water wash and subsequent addition of 0.5% HCl. The section was washed with water (~20 s), then treated with 0.05% ammonia water (~10 s). The section was thoroughly washed with water and stained with Ventana HE 600 eosin solution (lot no. H30444, Roche, Tucson, AZ, USA). After 1 min, the stained section was washed with ethanol. Finally, the air-dried section was visualized under a microscope (Nikon, Tokyo, Japan).

Lipid deposition in the liver was evaluated using Oil Red O (ORO) staining [64]. In brief, the liver section was covered with the ORO solution. After a 5 min incubation at 60 °C, the stained section was washed with water and visualized under a microscope.

### 4.9. Immunohistochemical (IHC) Analysis, Dihydroethidium (DHE), and Cellular Senescence Staining

The IL-6 level in the liver was determined by immunohistochemical staining. The liver section (7 μm thick) was rinsed with PBS and then treated with 3% H_2_O_2_ for 10 min, followed by PBS washing. The specimen was blocked with 5% normal goat serum for 30 min, followed by the addition of 250×-diluted IL-6-specific monoclonal antibodies (mouse IgG, NB600-1131, Novus Biochemicals, Centennial, CO, USA). The sample was incubated overnight (~14 h) in a cool, moist environment. Afterwards, the sample was washed with PBS. Subsequently, horseradish peroxidase (HRP) conjugated anti-IL-6 antibody (goat anti-mouse IgG, K4001, Dako, Glostrup, Denmark) was added. After 45 min incubation at RT, the sample was washed with PBS. Finally, the section was developed using the 3,3′-diaminobenzidine (DBA) chromogenic substrate (K3468, Dako, Glostrup, Denmark). The section was visualized under the microscope to detect the IHC-stained area and quantified using ImageJ software (https://image.net/ij, version 1.54s, assessed on 7 April 2026).

Oxidative stress in the tissue section was quantified by DHE fluorescence staining. The tissue section was covered with 200 μL of 30 μM DHE solution and incubated in the dark for 5 min. Following thorough washing with water, the section was visualized under a fluorescence microscope at an excitation wavelength of 585 nm and an emission wavelength of 615 nm. Finally, the images were processed in ImageJ software (version 1.54s, https://imagej.net/ij; assessed in 7 April 2026) to quantify DHE fluorescence intensity.

Senescence-associated β-galactosidase (SA-β-gal) staining was performed to detect senescent-positive cells in liver and kidney sections. The tissue section (7 μm thick) was covered with 0.1% X-gal (5-bromo-4-choloro-3-indolyl-β-D-galactopyranoside) solution for 16 h in a moist and cool atmosphere. Afterwards, the section was washed and visualized under a microscope to detect blue-stained senescent-positive cells.

### 4.10. Masson’s Trichrome Staining

Masson’s trichrome staining was performed to detect intestinal tissue fibrosis using the previously described methodology [72]. In brief, intestinal tissue (7 μm thick) was covered with Weigert’s iron hematoxylin solution [prepared by mixing equal proportions of solution A (4 g hematoxylin in 200 mL of 80% ethanol) and solution B (8 g FeCl3 in 190 mL distilled water and 2 mL of HCl)]. After 5 min of incubation in the dark, the section was washed three times with distilled water and subsequently immersed in Bedrich scarlet–acid fuchsin solution (prepared by dissolving 2.25 g Bedrich scarlet and 0.25 g acid fuchsin in 250 mL distilled water containing 2 mL of glacial acetic acid). After 5 min, the section was washed three times with distilled water and subsequently immersed in 1% phosphomolybdic acid. Following 2 min incubation, the section was treated for 5 min in 1.8% aniline blue (made by mixing 4.5 g of aniline blue in 250 mL of distilled water containing 4.5 mL of glacial acetic acid). The section was washed with distilled water, followed by 30 s treatment with 1% acetic acid. The section was finally rinsed in water and visualized under the microscope (Nikon, Tokyo, Japan).

### 4.11. Embryo-Producing Ability, Survival of Embryos and Developmental Defects

For the determination of the embryo production ability, zebrafish (male and female) from each tank of the specified group were used. From each tank, one male and two females were selected and transferred to a breeding tank to assess embryo production [31]. In the breeding tank, male and female zebrafish were separated overnight from each other using a physical divider. In the morning, the separated male and female zebrafish were allowed to mate uninterrupted for 30 min in the dark. Afterwards, the embryos produced were collected, counted, and transferred into a 0.01% sea salt solution containing 0.1 mg/mL methylene blue. The embryos were monitored until 72 h post-fertilization to assess survival. Morphological changes in developing embryos were assessed at 72 and 144 h post-fertilization, following the guidelines of the OECD (2019) [73]. Three independent breeding events (corresponding to the three different tank/group) were conducted for each experimental group. The embryos produced from each breeding event were collected for embryo survival analysis. Therefore, each tank/breeding event was considered an experimental unit, with three independent biological replicates per experimental group for both embryo production and embryo survival analysis.

### 4.12. Statistical Analysis

The experiments were carried out at least in triplicate, and the results are depicted as the mean value ± standard error of the mean (SEM). To analyze the statistical differences among multiple groups, one-way ANOVA with Tukey’s post hoc test was performed using the Statistical Package for the Social Sciences (SPSS, version 29, Chicago, IL, USA). The paired *t*-test was conducted to compare bivariate data from the same group at two different time points. Before performing the ANOVA, the normality of the data was examined using the Kolmogorov–Smirnov test.

## 5. Conclusions

This study concludes that the Poli+Ator combination has a significantly higher beneficial effect than either component alone in counteracting HCHG-induced dyslipidemia. Individually, Ator supplementation showed no response in reducing glucose levels, promoting tail fin regeneration, or protecting organs. However, Ator, in combination with Poli (Poli+Ator), substantially lowers blood glucose levels, promotes tail fin regeneration, and protects the liver and kidneys from damage. Also, Poli+Ator protects HCHG-impaired intestinal fibrosis and reproductive health. In conclusion, Poli+Ator showed an effective role in countering metabolic stress and related organ damage. However, further investigations in a distinct mammalian model and clinical studies are required to assess the potency and safety of the Poli+Ator combination for human application.

## Data Availability

The original contributions presented in this study are included in the article/Supplementary Material. Further inquiries can be directed to the corresponding author.

## Supplementary Materials

The following supporting information can be downloaded at: https://www.mdpi.com/article/10.3390/ijms27177687/s1.
