# Supplementary material for: Protective Effects of Policosanol and Atorvastatin Co-Supplementation Against High-Cholesterol and High-Galactose Diet-Induced Metabolic and Organ Dysfunction in Zebrafish
Source: Int J Mol Sci. 2026 Aug 27;27(17):7687. doi: 10.3390/ijms27177687 (PMC13566858; doi:10.3390/ijms27177687)
Supplement: Supplementary file 1 [file ijms-27-07687-s001.zip › ijms-4483713-supplementary.pdf]

Supplementary materials

Supplementary Table S1

RAYDEL
Health Confidence

Raydel Australia Pty Ltd
Level 1, building 1, 9-15 Chilvers Rd.
Thornleigh NSW 2120 Australia
Tel +61 2 9480 1300
Fax +61 2 9480 1399
A.B.N. 45 054 555 903
www.raydel.com.au
info@raydel.com.au

Product name: Policosanol

Batch #: 310030324

Date of Manufacture: 12/03/2024

Parameter	Results	Approved Limits
Color	Complies	Off white to cream
**Identity and Purity***		
1-tetracosanol (C_{24})	0.05 %	0.00 – 2.0 %
1-hexacosanol (C_{26})	3.34 %	3.0 –10.0 %
1-heptacosanol (C_{27})	0.82 %	0.1 - 3.0 %
1-octacosanol (C_{28})	60.02 %	60.0 –70.0 %
1-nonacosanol (C_{29})	0.55 %	0.1- 2.0 %
1-triacontanol (C_{30})	14.96 %	10.0 – 15.0%
1-dotriacontanol (C_{32})	8.24 %	5.0 - 10.0 %
1-tetratriacontanol (C_{34})	2.39 %	0.1 – 5.0 %
**Total (Purity*)**	**90.37 %**	**≥ 90 %**
**Other quality specifications**		
**Melting temperature**	81.3-83.0 °C	78.0 – 83.0 °C
**Loss on drying**	0.54 %	≤ 1.0 %
**Residue of ignition**	0.72 %	≤ 0.85 %
**Heavy metals (Pb, Cd, Hg)**	<0.000015 %	≤ 0.001 %
**Sodium content**	86.34 ppm	≤ 100 ppm
**Potassium content**	2905.41 ppm	≤ 4500 ppm
**Residual solvents**		
**Acetone**	≤ 0.03	≤ 0.03 g/kg
**Hexane**	≤ 0.005	≤ 0.005 g/kg
**Microbiological content**		
**Total Aerobic Microbial Count**	≤10	≤10^3 per g
**Yeast and mould**	≤10	≤10^2 per g
**Enterobacteria or Coliform count**	≤10	≤10^2 per g
***Staphylococcus aureus, Pseudomonas aeruginosa, Escherichia* coli, Candida albicans**	Absent	Absent in 1 g
Salmonella sp	Absent	Absent in 10 g
Observations:		
**References:**	* Manufacturer GC validated method, purity expressed as the total of high molecular weight alcohols	

Note about storage conditions: No special storage conditions are required. The substance has a shelf life of 5 years stored under ambient conditions of climatic Zones IV or II, as demonstrated in stability studies performed according to ICH guidelines.

Approved (_X_) Released (_X_) Rejected (___)

This COA is it reproduced from supplier's COA

Table S1: Certificate of analysis and the composition of the policosanol used.

Supplementary Table S2

Original certificate of water quality

분석과학을 선도하는 기업
KIRIM (주)기림생명과학원
KiRim Institute of Life Science Co.,Ltd.

받 음: 대구 동구 동내로 76 (동내동)
한국메디벤처센터 지하2층 관리실 귀하

41061

수질검사성적서

수신: 한국메디벤처센터
제목: 먹는물(저수조수) 수질검사성적서 발급

발 급 일: 2025년 5월 2일
발급번호: KWCT3379961
발 신: (주)기림생명과학원

「먹는물 수질기준 및 검사 등에 관한 규칙」 제3조제2항에 따라 다음과 같이 먹는물 수질검사성적서를 발급합니다. 이 성적서는 의뢰인이 제시한 시료에 대한 결과이며 검사목적 이외에는 사용할 수 없습니다.

1. 시료 내용

접수번호	12506438	접 수 일	2025년 4월 28일
의 뢰 인	한국메디벤처센터		
시 료 명	저수조수		
검사목적	수도법(저수조수 수질검사)		
채수장소	대구 동구 동내로 76 (동내동)		
시료채취	기술인력채취 (주)기림생명과학원 성기현 채수일시 : 2025.04.28		
비 고	판정은 수도법 시행규칙 제22조4의⑤항 및 ⑦항에 따른 먹는물 수질기준 및 검사등에 관한 규칙[별표1]에 의거합니다.		

2. 수질검사 결과

검사항목	기준	검사결과
탁도	0.5 NTU 이하	0.16
수소이온농도	5.8 ~ 8.5	7.3
잔류염소	0.1 ㎎/L이상 4.0 ㎎/L이하	0.18
일반세균	100 CFU/mL이하	0
총대장균군	불검출/100mL	불검출
분원성대장균군	불검출/100mL	불검출
종합결과	적합	

• 탁도(등급외 성능),잔류염소(1등급)는 간이측정기를 사용하여 측정함.

(주)기림생명과학원 대표이사

- 환경과 생명을 소중히 하는 아름다운 기업 -

Certificate of water quality (translated in English)

Drinking Water Quality Test Report
Recipient: Korea Mediventure Center
B2 Management Office
76 Dongnae-ro, Dong-gu, Daegu (Dongnae-dong), 41061
Issued by: Kirim Life Science Co., Ltd.
Issue Date: May 2, 2025
Report Number: KWCT3379961
Subject: Drinking Water (Reservoir Water) Quality Test Report

Basis of Issuance
This report is issued in accordance with Article 3, Paragraph 2 of the *Regulations on Drinking Water Quality Standards and Testing*.
It presents the results of the sample provided by the client. The report must not be used for purposes other than water quality testing.

1. Sample Information
•**Receipt Number:** 12506438
•**Receipt Date:** April 28, 2025
•**Client:** Korea Mediventure Center
•**Sample Name:** Reservoir Water
•**Purpose of Test:** Waterworks Act (Reservoir Water Quality Test)
•**Sampling Location:** 76 Dongnae-ro, Dong-gu, Daegu (Dongnae-dong)
•**Collected by:** Technical staff (Seong Ki-hyun, Kirim Life Science Co., Ltd.)
•**Sampling Date/Time:** April 28, 2025
•**Remarks:** Evaluation conducted in accordance with Article 22, Paragraph 4 of the Enforcement Rules of the Waterworks Act and Annex 1 of the *Regulations on Drinking Water Quality Standards and Testing*.

2. Test Results

Test Item	Standard	Result
Turbidity	≤ 5 NTU	0.16
pH (Hydrogen Ion)	5.8 – 8.5	7.3
Residual Chlorine	0.1 – 4.0 mg/L	0.18
General Bacteria	≤ 100 CFU/mL	0
Total Coliform Group	Not detected / 100 mL	Not detected
Fecal Coliform Group	Not detected / 100 mL	Not detected

Comprehensive Result: Suitable (Compliant)

Note: Turbidity (non-graded performance) and residual chlorine (Grade 1) were measured using a simplified measuring device.

Certification
Issued by: **Kirim Life Science Co., Ltd.** Representative Director
- A beautiful company that values environment and life -

Table S2: Certificate of water quality analysis.

Section S1.

1. Method to quantify plasma lipoproteins and hepatic function biomarkers

The plasma total cholesterol (TC), triglyceride (TG), and high-density lipoprotein cholesterol (HDL-C) were determined using commercial assay kits (TC, AM 202-K, TGs, AM 157-K, and HDL-C, AM 203-K) Asan Pharmaceutical, Hwasung, Republic of Korea) as per the

method suggested by the suppliers. In brief, 5 μL plasma was mixed with 200 μL reaction mixture (supplied with a commercial assay kit) for the TC analysis. The content was incubated at 37°C for 10 min, resulting in a red-colored product quantified by adsorption at 490 nm (Microplate reader, iMark™ Bio-Rad, Hercules, CA, USA).

Similarly, 5 μL plasma was mixed with a 200 μL of TGs-specific reaction mixture (supplied with a commercial assay kit) for TGs analysis. The content was incubated for 10 min at 37°C, and the formed colored product was quantified by taking adsorption at 490 nm.

For HDL-C analysis, plasma was mixed in an equal ratio with the separation solution (supplied with a commercial assay kit), followed by centrifugation at 3,000 rpm for 10 min at 25°C. The supernatant (20 μL) was collected and blended with a 200 μL reaction mixture (supplied with a commercial assay kit). After 10 min incubation at 37°C, red color intensity corresponding to HDL-C was quantified by taking absorption at 490 nm (Microplate reader, iMark™ Bio-Rad, Hercules, CA, USA).

The LDL-C level was quantified using the Friedewald equation:

$$LDL\text{-}C = TC - HDL\text{-}C - (TG/5)$$

The commercial diagnostic kit (AM 102-K and AM 103-K, Asan Pharmaceutical, Hwasung, Republic of Korea) was used to quantify aspartate transaminase (AST) and alanine transaminase (ALT) levels in the plasma, following the instructions suggested by the manufacturers. Briefly, 5 μL of plasma was combined with 250 μL of either AST or ALT-specific solution, as supplied in the diagnostic kit. Following a 30 min incubation for AST or 60 min incubation of ALT at 37°C, the mixture was then blended with 250 μL of the respective coloring reagent (AST or ATL-specific, provided in the diagnostic kit). After a subsequent 20 min incubation at room temperature, 250 μL of 0.4 N NaOH was introduced to halt the reaction. Finally, the AST and ATL were quantified by measuring absorbance at 490 nm (Microplate reader, iMark™, Bio-Rad, Hercules, CA, USA).

Section S2.

2. Malondialdehyde (MDA), sulfhydryl group, ferric ion reduction (FRA) activity and paraoxonase (PON) activity

The blood malondialdehyde (MDA) level was quantified by mixing plasma sample (20 μL, equivalent to 1 mg/mL protein) with trichloroacetic acid (50 μL, 0.2 mg/μL, pH 1.4) and thiobarbituric acid (100 μL, 6.7 μg/μL, pH 2.3). Following a 10-min incubation at 95 °C, the absorbance at 560 nm was recorded.

The sulfhydryl group was quantified by mixing 60 μL of plasma (1 mg/mL protein) with 60 μL of 5,5′-dithio-bis-(2-nitrobenzoic acid) (DTNB) (4 mg/mL). After 12 hr incubation at room temperature, absorbance 412 nm was determined, and sulfhydryl groups were quantified utilizing 13,600 M^{-1} cm^{-1} extinction coefficient (e) of DTNB.

To assess ferric ion reduction (FRA) capacity, 20 μL of the plasma (1 mg/mL equivalent protein) was mixed with 180 μL of FRA reagent (prepared by blending 10 mL of acetate buffer (0.2M, pH 3.6) with 1.25 mL each of 2,4,6-tripridyl-S triazin (10 mM, pH 1.9) and

$FeCl_3$ (20 mM, pH 2.1). After incubating the mixture at RT for 60 min, absorbance was measured at 593 nm. The results were quantified in µM ferric equivalents based on a ferrous sulfate standard curve.

For paraoxonase activity 20 µL of plasma (1 mg/mL equivalent protein) was mixed with 180 µL of buffer (pH 8.3) [Tris-HCl (90 mM), NaCl (3.6 mM), $CaCl_2$ (90 mM)] containing the paraoxon-ethyl substrate (0.55 M). After 60 min incubation at 25°C, an absorbance (415 nm) was recorded using Microplate reader (Model, iMark™ S.N 21275, Bio-Rad, Hercules, CA, USA) to quantify the production of *p*-nitrophenol, a hydrolysis product of paraoxon-ethyl. Results are expressed as µU/L/min employing the extinction coefficient 17,000 $M^{-1}cm^{-1}$ for *p*-nitrophenol.
